# A Long-Term Cultivation of an Anaerobic Methane-Oxidizing Microbial Community from Deep-Sea Methane-Seep Sediment Using a Continuous-Flow Bioreactor

**DOI:** 10.1371/journal.pone.0105356

**Published:** 2014-08-20

**Authors:** Masataka Aoki, Masayuki Ehara, Yumi Saito, Hideyoshi Yoshioka, Masayuki Miyazaki, Yayoi Saito, Ai Miyashita, Shuji Kawakami, Takashi Yamaguchi, Akiyoshi Ohashi, Takuro Nunoura, Ken Takai, Hiroyuki Imachi

**Affiliations:** 1 Department of Subsurface Geobiological Analysis and Research (D-SUGAR), Japan Agency for Marine-Earth Science and Technology (JAMSTEC), Yokosuka, Kanagawa, Japan; 2 Department of Environmental Systems Engineering, Nagaoka University of Technology, Nagaoka, Niigata, Japan; 3 Institute for Geo-resources and Environment, National Institute of Advanced Industrial Science and Technology (AIST), Tsukuba, Ibaraki, Japan; 4 Department of Construction Systems Engineering, Anan National College of Technology, Anan, Tokushima, Japan; 5 Department of Social and Environmental Engineering, Hiroshima University, Higashihiroshima, Hiroshima, Japan; 6 Research and Development Center for Marine Biosciences, JAMSTEC, Yokosuka, Kanagawa, Japan; Laval University, Canada

## Abstract

Anaerobic oxidation of methane (AOM) in marine sediments is an important global methane sink, but the physiological characteristics of AOM-associated microorganisms remain poorly understood. Here we report the cultivation of an AOM microbial community from deep-sea methane-seep sediment using a continuous-flow bioreactor with polyurethane sponges, called the down-flow hanging sponge (DHS) bioreactor. We anaerobically incubated deep-sea methane-seep sediment collected from the Nankai Trough, Japan, for 2,013 days in the bioreactor at 10°C. Following incubation, an active AOM activity was confirmed by a tracer experiment using ^13^C-labeled methane. Phylogenetic analyses demonstrated that phylogenetically diverse *Archaea* and *Bacteria* grew in the bioreactor. After 2,013 days of incubation, the predominant archaeal components were anaerobic methanotroph (ANME)-2a, Deep-Sea Archaeal Group, and Marine Benthic Group-D, and *Gammaproteobacteria* was the dominant bacterial lineage. Fluorescence *in situ* hybridization analysis showed that ANME-1 and -2a, and most ANME-2c cells occurred without close physical interaction with potential bacterial partners. Our data demonstrate that the DHS bioreactor system is a useful system for cultivating fastidious methane-seep-associated sedimentary microorganisms.

## Introduction

The microbially mediated anaerobic oxidation of methane (AOM) in marine sediments is a globally important microbial process in carbon cycling [1]. AOM-associated microorganisms have been extensively studied using biogeochemical and microbiological approaches. A consensus in the field of AOM studies is that euryarchaeal anaerobic methanotrophs (ANMEs) oxidize methane either solely or in syntrophic association with deltaproteobacterial sulfate-reducing bacteria (SRB) [2]. ANMEs are phylogenetically closely related to known methanogenic *Archaea* and can be classified into three distinct phylogenetic lineages called ANME-1, -2, and -3 [2]. Several groups of SRB partners have been identified, including, SEEP-SRB1, SEEP-SRB2 (also known as the Eel-2 group), HotSeep-1, seepDBB and *Desulfobulbus* relatives [3]–[7]. In addition, some previous reports have suggested the possible involvement of other uncharacterized microorganisms in AOM [8]–[12]. Pernthaler *et al*. [13] found that not only deltaproteobacterial SRB partners but also uncharacterized bacteria belonging to *Alpha-* and *Beta-proteobacteria* formed aggregates with ANME-2c cells. Metagenomic, metatranscriptomic, and metaproteomic studies have indicated that AOM is catalyzed by a reverse methanogenesis pathway [13]–[17]. However, neither the ANMEs nor their potential syntrophic partners have been isolated, and thus their detailed physiological properties remain poorly understood.

To gain a deeper understanding of carbon cycling in methane-seep sediments, the cultivation of AOM-associated microbial communities is a significant challenge. Several research groups have employed continuous-flow bioreactor systems for the activation and enrichment of AOM microbial communities [18]–[23]. In addition to the bioreactor enrichments, a few enrichment cultures have been obtained using batch-type cultivation methods, following long-term incubation [4], [7], [24]. However, due to the extremely slow growth rate of AOM microbial communities (i.e., the estimated doubling time is several months) [20],[24]–[26], the cultivation of AOM microbial communities is laborious, and knowledge of AOM enrichment cultures remains limited.

**Figure 1:**
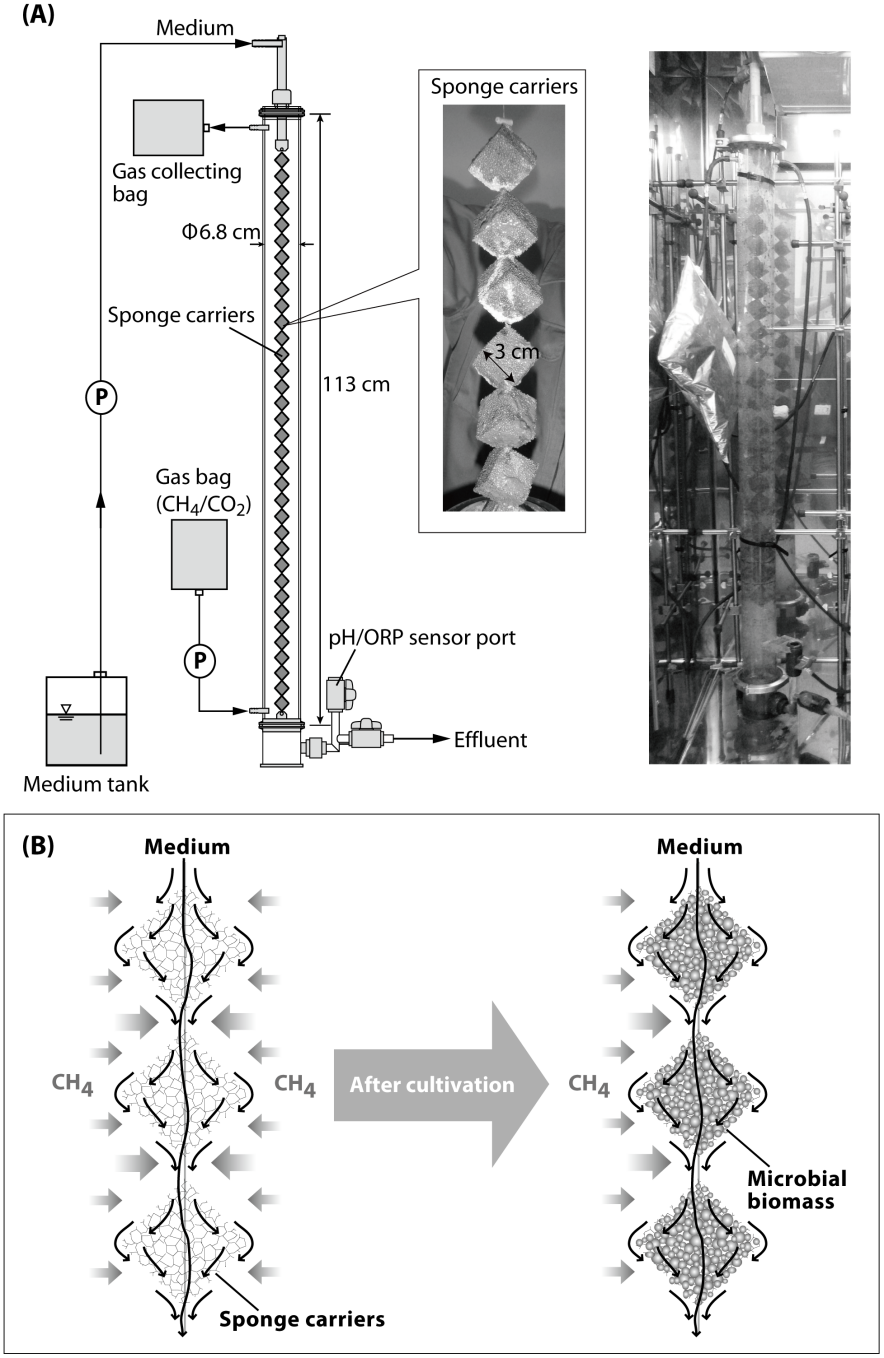
The DHS bioreactor system. (A) Schematic diagram and photographs of the DHS bioreactor used in this study. (B) The concept of cultivation of an AOM microbial community using the DHS bioreactor. Sponge carriers hang freely, suspended with string, in gaseous methane. Thus, the gaseous methane effectively diffuses not only to the surface but also inside the sponge carriers as the seawater medium flows down into the sponge carriers. The pore space in the sponge carriers serves as the habitat for microbial life.

To effectively cultivate AOM-associated microorganisms, we employed a continuous-flow bioreactor technique. The bioreactor used in this study is a down-flow hanging sponge (DHS) bioreactor (Fig. 1) originally developed for municipal wastewater treatment [27]–[28]. A distinctive feature of the DHS bioreactor is the use of polyurethane sponges, providing an enlarged surface for microbial habitats and an increased cell residence time. In addition, the sponge carriers are not submerged in the medium but are hanging freely in gaseous substrates (e.g., methane), and thus the gaseous substrates effectively diffuse inside the sponge carriers as the influent medium flows through them. Moreover, continuous flow allows the outflow of metabolic products such as hydrogen sulfide (in the case of sulfate-dependent AOM), which may inhibit microbial growth if allowed to accumulate. These properties of DHS bioreactors allow slow-glowing microorganisms to thrive and yield a greater biomass than that observed when other bioreactor systems are used [29].

In this study, deep-sea methane-seep sediment collected from the Nankai Trough, Japan was incubated anaerobically for 2,013 days in a newly designed DHS bioreactor system, in order to cultivate AOM-associated microorganisms. Following long-term incubation in the bioreactor, an AOM microbial community that consisted of ANMEs and phylogenetically diverse yet-to-be-cultured microorganisms was successfully enriched from the methane-seep sediment.

## Materials and Methods

### Ethics statement

The location for sample collection was not privately owned or protected in any way and no specific permits were required for the described field studies and sample collection. The field studies did not involve any endangered or protected species.

### Sediment core sample

An active methane-seep sediment core (949C3) was collected from the Omine Ridge, Nankai Trough off Kumano area, Japan (33° 7.23′ N, 136° 28.67′ E), at 2,533 m below the sea surface, via the manned submersible ‘*Shinkai 6500*’ (cruise YK06-03, dive no. 6K949, 6 May 2006). The sediment consisted of blackish-gray, sandy silt and contained hydrogen sulfide. The sediment core was 25 cm in length and was subsampled using sterilized top-cut syringes and spatulas at 5-cm intervals on board. The subsampled sediments were used for interstitial water geochemical analysis and for culture-independent molecular analyses. The geochemical data showed that the sediment contained more than 5.4×10^2^ µmol kg^−1^ methane throughout the sediment core and that sulfate concentrations in the core decreased as the depth increased (Toki *et al*., personal communication). The results of the culture-independent molecular analyses (i.e., quantitative real-time PCR and 16S rRNA gene tag-sequencing) have been reported by Nunoura *et al*. [30]. In this study, we used the sediment that remained after the subsampling. The sediment sample was stored anaerobically under nitrogen gas at 4°C in the dark, until further experiments were performed.

### DHS bioreactor and incubation of sediment

A schematic diagram of the DHS bioreactor is shown in Fig. 1. The bioreactor was constructed from a closed polyvinyl chloride (PVC) column (interior volume of 4.4 L) with polyurethane sponge cubes (3 cm×3 cm×3 cm, pore size of 0.83 mm) as the carrier material for cultivating the microbial cells. The 22 sponge cubes were hung vertically using a nylon string. The total volume of the sponges was 0.59 L, and this value was used for calculating the hydraulic retention time (HRT). The inoculum sediment was mixed with an anaerobic medium (described below) and the sponge carriers were soaked with the mixed sediment slurry. This inoculation procedure was performed in a cold room maintained at 4°C, and the sediment slurry and PVC column were constantly flushed with nitrogen gas. Following the inoculation, the PVC column was tightly closed and installed in an incubator (M-600FN, TAITECH, Koshigaya, Japan) in the dark at 10°C. The composition of the supplied medium was as follows (L^−1^): Daigo’s artificial seawater SP for marine microalgae medium (Nihon Pharmaceutical, Tokyo, Japan), 36 g; NH_4_Cl, 0.54 g; KH_2_PO_4_, 0.14 g; NaHCO_3_, 2.5 g; glucose, 0.01 g; Na_2_S·9H_2_O, 0.11 g; trace element solution [31], 1 mL; vitamin solution [32], 1 mL; and titanium (III)-nitrilotriacetic acid solution [33], 5 mL. The medium contained 24.7 mM sulfate. The medium was purged with nitrogen gas, and the pH was adjusted to 7.5. A total of 5 L of media was prepared at a time. The medium was stored at 5°C and was supplied into the bioreactor through the top inlet port using a peristaltic pump (Masterflex L/S tubing pump 7550-50, Cole-Parmer, Vernon Hills, IL, USA) and Viton tubing (Cole-Parmer). The medium then flowed down, passing through the sponge carriers by gravity, and was finally pumped out of the PVC column. The HRT in the bioreactor was set at 20 h. A gas mixture of methane and carbon dioxide (95∶5, vol./vol.) was prepared in an aluminum bag (AAK-5, ASONE, Osaka, Japan) and supplied to the lower part of the bioreactor. The medium and CH_4_/CO_2_ gas were supplied intermittently at 1 min/9 min (on/off), regulated by an automatic on/off timer (FT-011, Tokyo Glass Kikai Co. Ltd., Tokyo, Japan) connected to the peristaltic pump. The effluent gas was collected in an aluminum bag, via Viton tubing, from the top portion of the bioreactor. The bioreactor was operated under atmospheric pressure.

### Chemical analysis and sampling from the DHS bioreactor

The pH and oxidation-reduction potential (ORP) of the effluent medium were measured using a pH and redox electrode (InPro3250SG, Mettler-Toledo, Greifensee, Switzerland) connected to a modular transmitter (M700, Mettler-Toledo). The sulfate concentration of the influent/effluent medium was measured using a turbidimetric method (Hach Method 8051) by using subsamples diluted to 100-fold with Milli-Q water. For microbial community analyses, sediment slurry samples attached to the sponge carriers were sampled at 285, 903, 1,376, 1,529, 1,732, and 2,013 days of operation. At each sampling, several sponge carriers were randomly selected from 3 portions (upper, middle, and lower) of the bioreactor. The sediment slurry samples were removed from the selected sponges by manual rubbing in anaerobic seawater medium. Nitrogen gas was flushed during the sampling procedure.

### Measurement of potential AOM activity

A tracer experiment using ^13^C-labelled methane was used to estimate potential AOM activity, as described by Yoshioka *et al*. [34]. To measure activity, approximately 30 mL of the bioreactor incubation samples collected on day 1,529 was aliquotted into four 70-mL vials. After flushing with nitrogen gas, the vials were sealed with butyl rubber stoppers and aluminum crimp seals. During the incubation procedure, all samples were maintained under anaerobic conditions; this was confirmed by the clear color of the resazurin in the samples. Thirty milliliters of non-labeled methane taken from a tank [δ^13^C = −36.6 ‰ (PDB)] was injected into 2 of the vials with a glass syringe, while 27 mL of the non-labeled methane and 3 mL of 100% ^13^C-labeled methane (Sigma-Aldrich/ISOTEC, St. Louis, MO, USA) were injected into the other 2 vials. All the vials were incubated at 10°C in the dark. To monitor the differences in the stable carbon isotope compositions of dissolved inorganic carbon (DIC), 1 mL of solution was collected from each vial after 1, 14, 29, and 42 days of incubation. The stable carbon isotope compositions of the DIC in the solutions were measured using a Thermoquest-Finnigan Gas Bench linked to a DeltaPlus XL mass spectrometer (Thermo Finnigan Inc., Austin, TX, USA). We calculated the ^13^C-enrichment of the DIC in the ^13^CH_4_-amended vials relative to the non-amended vials and estimated the methane oxidation rate by calculating the ^13^C-enrichment in the total DIC.

### Nucleic acid extraction, PCR, cloning, and phylogenetic analysis

DNA extraction, PCR amplification, clone library construction, and sequencing were performed as described previously [35]. The PCR primers used in this study are shown in Table S1. The PCR primer pairs Arch21F/Ar912r and EUB338F/1492R were used for the construction of 16S rRNA gene-based archaeal and bacterial clone libraries, respectively. For the construction of the *mcrA* gene-based clone library, we used the primer pair MLf/MLr. PCR was performed under the following conditions: initial denaturation at 95°C for 9 min, followed by 20 to 35 cycles of denaturation at 95°C for 40 s, annealing at 50°C for 30 s, and extension at 72°C for 1 min. To reduce possible bias caused by PCR amplification, PCR products obtained following the minimal number of PCR cycles, ranging from 20 to 35 cycles at five-cycle intervals, were used.

Total RNA extraction was performed immediately following sampling from the bioreactor, using a previously described method [36]. The remaining DNA was digested with RNase-free DNase I (Promega, Madison, WI, USA). The absence of genomic DNA contamination in the RNA extracts was confirmed by PCR, using the same PCR primer pairs. The concentration of RNA was quantified spectrophotometrically using a Quant-iT RNA assay kit (Invitrogen, Carlsbad, CA, USA). Reverse transcription (RT)-PCR was performed using a SuperScript III One-Step RT-PCR System with Platinum Taq DNA polymerase (Invitrogen), according to the manufacturer’s instructions. The RT-PCR primers and subsequent procedures were identical to those employed in the 16S rRNA gene-based clone analysis presented above.

Recovered clone sequences were classified into phylotypes using a threshold of 97% sequence identity. The representative sequences of the phylotypes were subjected to BLASTN analysis [37]. 16S rRNA gene sequence-based phylogenetic tree construction was performed using the neighbor-joining method with the Jukes-Cantor correction, as described previously [38]. A deduced McrA amino acid sequence-based phylogenetic tree was constructed using the neighbor-joining method, implemented in the ARB program, version 5.2 [39] using 137 amino acid positions and PAM distance correction. The sequences reported in this study have been deposited in the GenBank/EMBL/DDBJ database under the accession numbers AB831260–AB831537.

### Statistical analyses

Chao1 species richness and the Shannon diversity index were calculated using the EstimateS software, version 8.2 (http://viceroy.eeb.uconn.edu/estimates/). Clone library coverage was calculated using the equation [1−(*n*_1_/*N*)]×100, where *n*_1_ is the number of single-occurrence phylotypes within a library and *N* is the total number of clones in the library [40]. Evenness was calculated using the equation *H*/ln *R,* where *H* is the Shannon diversity index and *R* is the number of phylotypes observed within a library [41]. Rarefaction curves were calculated using the Analytic Rarefaction software, version 2.0 (http://www.huntmountainsoftware.com).

### Terminal restriction fragment length polymorphism (T-RFLP) analysis

Archaeal and bacterial 16S rRNA gene fragments for T-RFLP analysis were amplified by PCR using the primer pairs 5′ FAM-labeled Arch21F/Ar912r and 5′ FAM-labeled EUB338F/1492R, respectively. PCR was performed under the conditions described above. The PCR products were digested with HaeIII and HhaI (TaKaRa Bio Inc., Otsu, Japan) separately. The labeled fragments were analyzed by electrophoresis on an ABI 3130×l Genetic Analyzer (Applied Biosystems, CA, USA). GeneScan 1200 LIZ (Applied Biosystems) was used as a size standard.

### Quantitative real-time PCR

Quantitative real-time PCR of archaeal and bacterial 16S rRNA genes was performed with a 7500 Real-Time PCR System (Applied Biosystems) using a SYBR Premix Ex Taq II (Perfect Real Time) kit (TaKaRa Bio Inc.). For archaeal and bacterial 16S rRNA gene quantification, the primer pairs 340F/932R and EUB338F/907R, respectively, were used (Table S1). The reaction mixture for real-time PCR was prepared according to the manufacturer’s instructions. For the construction of template standards for each primer set, we used dilution series of 16S rRNA gene fragments of *Methanobacterium* sp. strain MO-MB1 [42] and *Escherichia coli* strain K12 (DSM 5911), which were obtained using the archaeal primer pair Arch21F/1492R and the bacterial primer pair 27F/1492R, respectively. These PCR products were used in each real-time PCR to calculate the 16S rRNA gene copy number. Template DNA was quantified spectrofluorometrically using a Quant-iT PicoGreen dsDNA Assay Kit (Invitrogen). The optimal PCR conditions, including the annealing temperature, were empirically determined for each primer pair. PCR was performed under the following conditions: initial denaturation at 95°C for 10 s, followed by 40 cycles of denaturation at 95°C for 5 s, 30 s of annealing (54°C for *Archaea*; 50°C for *Bacteria*), and extension at 72°C for 34 s. To verify the specificity of the real-time PCR assay, the PCR products were subjected to melting curve analysis (60–90°C) and subsequent gel electrophoresis. All assays were performed in triplicate.

### Fluorescence *in situ* hybridization (FISH)

Subsamples for FISH were fixed with 2% paraformaldehyde in anaerobic medium excluding glucose for 12 to 16 h at 4°C and stored in a 1∶1 mix of phosphate-buffered saline (PBS): ethanol at −20°C. The FISH samples used in this study were not dispersed by homogenization or ultrasonication prior to *in situ* hybridization procedures. The 16S rRNA-targeted oligonucleotide probes used in this study are listed in Table S2. The 5′ ends of the oligonucleotide probes were labeled with Alexa Fluor 488 or horseradish peroxidase (HRP). Standard FISH was performed according to previously described methods [43] with minor modifications. In brief, hybridizations were carried out in hybridization buffer (20 mM Tris–HCl [pH 7.2], 0.9 M NaCl, 0.01% sodium dodecyl sulfate, 1% [wt./vol.] blocking reagent [Roche Diagnostics, Mannheim, Germany], 0 to 65% formamide) with 0.5 µM of probe overnight at 46°C in the dark. The washing step was performed at 48°C for 15 min with washing buffer containing the same components as the hybridization buffer, except for the blocking reagent and probes. Catalyzed reporter deposition (CARD)-FISH [44] with HRP-labeled probes was performed based on a previously described method [45]. To inactivate endogenous peroxidase activity, the fixed samples were incubated in H_2_O_2_ solution (final concentration, 0.3% [vol./vol.] in methanol) for 30 min at room temperature. An HRP-labeled negative control probe, NON338, was used as the negative control. For the multi-color CARD-FISH using the probes ANME-2c-760 and EUB338, 0.01 M HCl solution (10 min at room temperature) was used for the inactivation of HRP in the initial hybridization. Hybridization conditions were controlled by varying the formamide concentrations in the hybridization and washing buffers. The hybridization conditions for six of the uncultured group-specific probes (ANME-1-350, ANME-2a-647, ANME-2c-760, MBGD-318, MBGB-380, and UncGam731) were determined by Clone-FISH [46]. The Clone-FISH samples were prepared as described in Kubota *et al*. [45]. The clonal sequences used for Clone-FISH were AB598074, AB598076, and AB831534 to AB831537. Four of the clonal sequences (AB831534 to AB831537) were obtained by 16S rRNA gene cloning with the primer pair Arch21F/1492R or 27F/1492R from the bioreactor incubation sample collected at day 903. The FISH samples were finally counterstained with 4′, 6-diamidino-2-phenylindole (DAPI; 1 µg mL^−1^ for 5 min at room temperature) after all of the *in situ* hybridization steps had been performed. An Olympus microscope (BX51F, Olympus, Tokyo, Japan) with a color CCD camera system (DP72, Olympus) was used for microscopic observations.

## Results

### DHS bioreactor operation and potential AOM activity in the bioreactor

We operated the DHS bioreactor (Fig. 1) at 10°C for a total of 2,013 days. During the initial period of bioreactor operation (∼365 days), the bioreactor could not adequately maintain reductive conditions, likely due to molecular oxygen contamination (Fig. S1A). When a medium containing resazurin, a redox indicator, was fed into the bioreactor, the medium at the top of the PVC column was very faintly pink in color. Therefore, to maintain more reductive conditions, we sealed all the joints in the tubing lines from the medium storage bottle to the bottom of the PVC column. Moreover, a tiny amount of glucose (0.01 g L^−1^) was added to the medium after day 365, with the intention that the contaminated molecular oxygen would be consumed by the activity of existing aerobic microorganisms in the bioreactor. Following these improvements, the ORP values of the effluent medium indicated that reductive conditions were maintained in the bioreactor (Fig. S1A). The average pH value of the effluent medium was 7.5 (SD±0.1, n = 1,441) (Fig. S1B). To confirm the occurrence of sulfate-dependent AOM reactions in the bioreactor, we measured the sulfate concentrations of the influent and effluent medium several times until 1,500 days using turbidimetric analysis. However, the sulfate concentrations in the effluent medium did not differ from those in the influent medium (i.e., approximately 25 mM sulfate). This result may be explained by the relatively higher sulfate loading rate (30 mmol L^−1^ day^−1^) compared to the AOM activity in the DHS bioreactor. We therefore conducted a tracer experiment using ^13^C-labelled methane to measure potential AOM activity using a sample collected at day 1,529. Throughout the experiment, an increase in the ^13^C content of the DIC was observed (Fig. S2A). In the case that only AOM occurred, the potential AOM rate would have been 375 nmol g-dry weight (dw)^−1^ day^−1^ (mean value, n = 2). However, recent studies have shown that AOM and methane production occur simultaneously in AOM systems [47]–[48]. If methane production had proceeded in the tracer experiment, the increased δ^13^C values of DIC could have included the effect of methane production activity [49]. Here, we focus on the δ^13^C values of DIC in the ^13^CH_4_-non-amended sample, which showed a decreasing trend in the incubation (Fig. S2B). The decreasing trend supports that ^12^C was enriched in the carbon dioxide/bicarbonate pool through AOM. Although the exact AOM/methane production activity ratio in the DHS bioreactor enrichment is unclear, the tracer experiment indicated that an active AOM microbial community had been established in the bioreactor.

### Abundance of archaeal and bacterial populations estimated by quantitative real-time PCR

**Figure 2:**
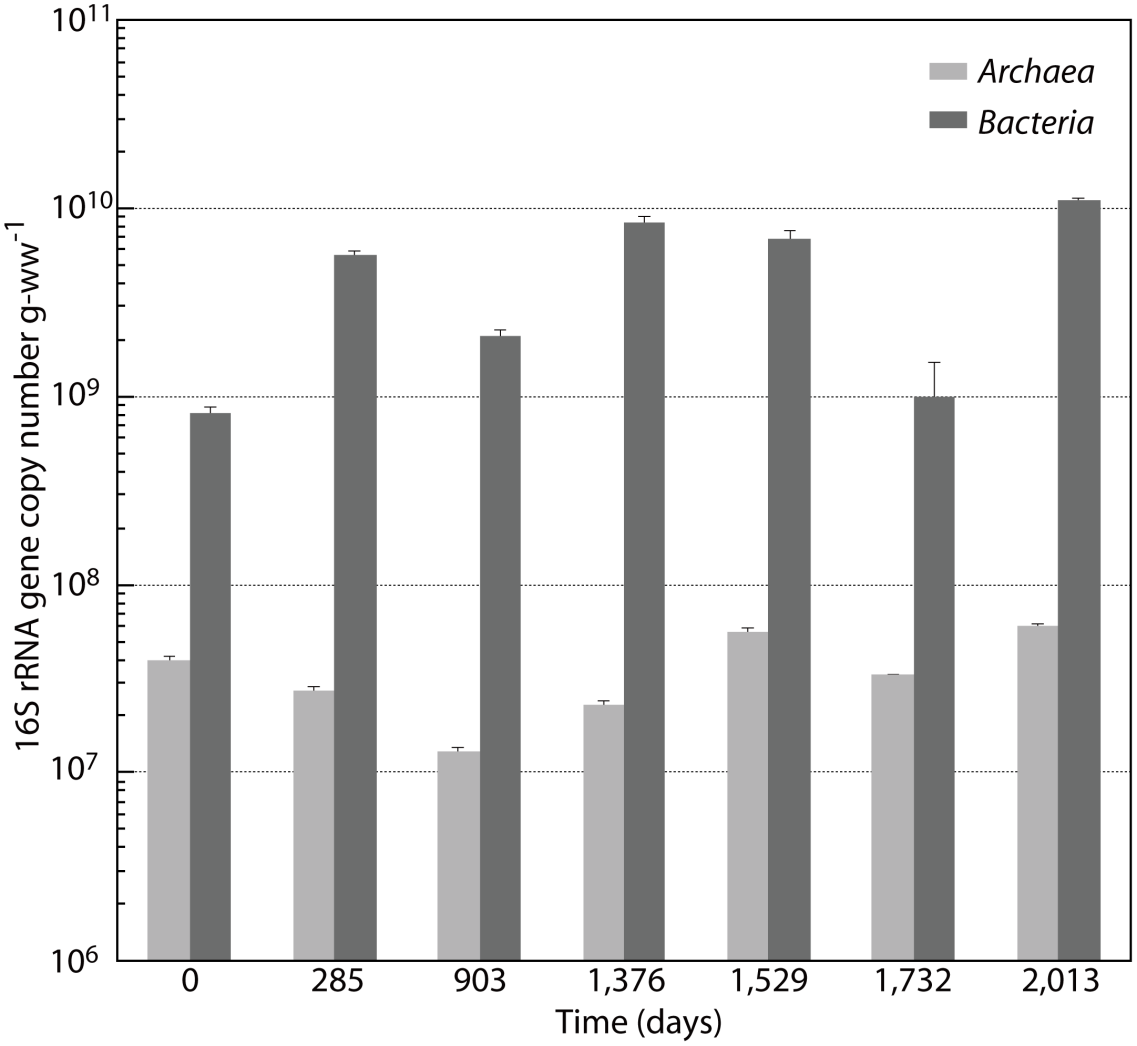
Archaeal and bacterial 16S rRNA gene copy numbers in the DHS bioreactor as determined by quantitative real-time PCR. Values are mean ± SD (n = 3).

To examine the changes in the abundance of archaeal and bacterial populations during the DHS bioreactor incubation, we quantified archaeal and bacterial 16S rRNA gene copy numbers (Fig. 2). In the inoculum sediment, the archaeal and bacterial 16S rRNA gene copy numbers were 4.0×10^7^ and 8.2×10^8^ copies g-wet weight (ww)^−1^, respectively. Following incubation in the bioreactor, the bacterial 16S rRNA gene copy number increased approximately tenfold, to 5.7×10^9^ copies g-ww^−1^ at day 285; the copy number was maintained at 10^9^ copies g-ww^−1^ for the duration of the reactor operation. In contrast, archaeal 16S rRNA gene copy numbers remained at 10^7^ copies g-ww^−1^ throughout the 2,013 days of incubation, although notable shifts were observed in the archaeal community, as mentioned below.

### Composition of the microbial community in the DHS bioreactor

**Figure 3:**
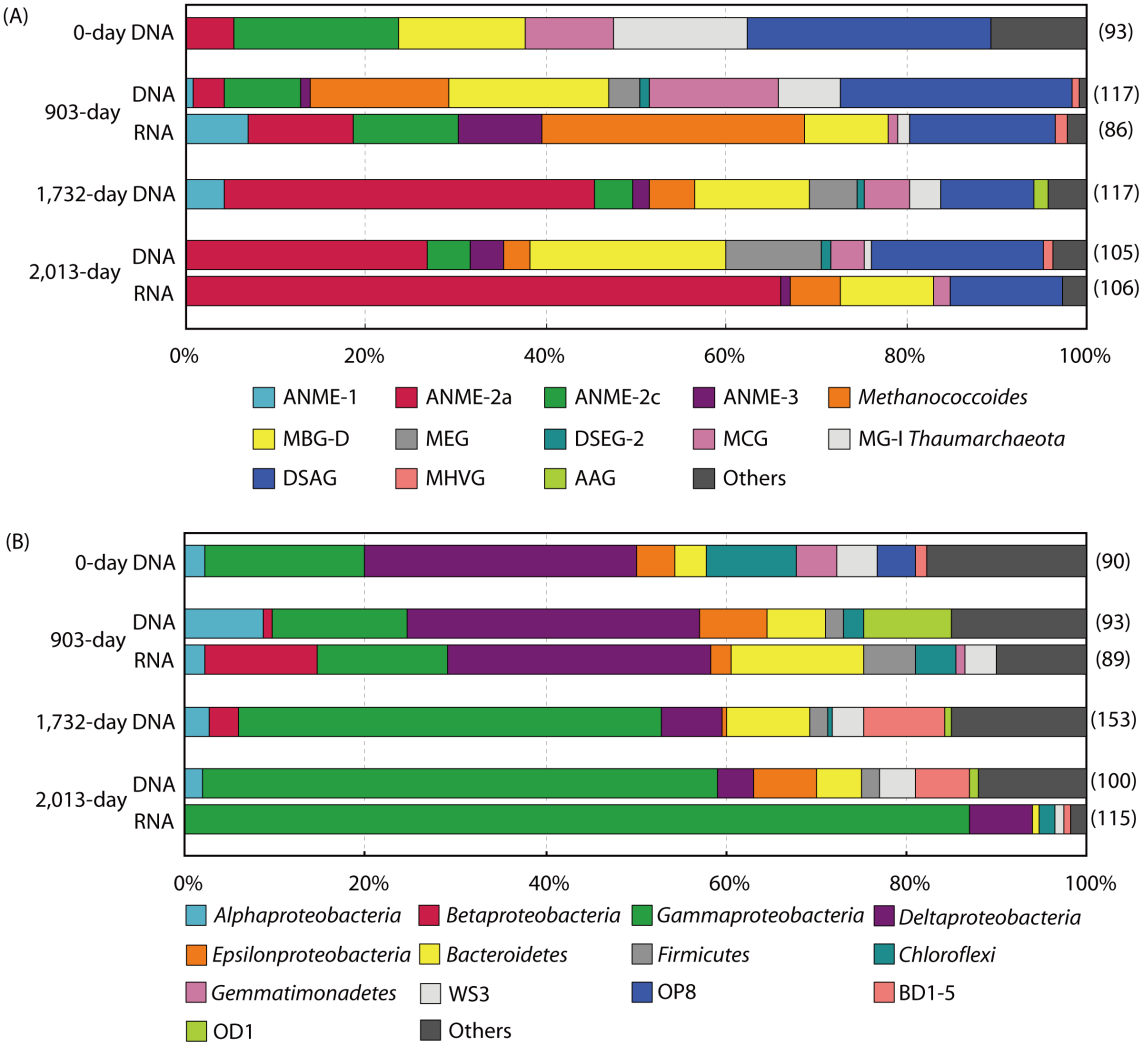
Microbial community structures based on 16S rRNA gene sequence-based clone libraries. Phylogenetic affiliations based on (A) archaeal and (B) bacterial 16S rRNA genes and 16S rRNA. The 16S rRNA clone libraries were constructed from the 903-day and 2,013-day samples only. The numbers in parentheses are the total number of sequenced clones.

To characterize the composition of the AOM microbial community and any shifts in the community during the DHS bioreactor incubation, we constructed clone libraries targeting archaeal and bacterial 16S rRNA genes for the bioreactor incubation samples (Fig. 3 and S3–S5). To identify the active microbial components in the bioreactor, we also constructed 16S rRNA clone libraries from the total RNA extracts prepared from the 903- and 2,013-day samples via RT-PCR.

The archaeal phylotypes detected in the inoculum sediment (i.e., the 0-day sample in Fig. 3A) were closely related to the sequences of ANME-2a, ANME-2c, Deep-Sea Archaeal Group (DSAG; also known as Marine Benthic Group-B), Marine Benthic Group-D (MBG-D), *Thermoplasmata* Group E2, Miscellaneous Crenarchaeotic Group (MCG), and Marine Group-I (MG-I) *Thaumarchaeota*, which are frequently observed in marine subsurface sediments. The relative abundance of ANME-related clones (i.e., ANME-2a and -2c) in the archaeal 16S rRNA gene clone library of the inoculum sediment was 23.7%. During incubation in the bioreactor, the archaeal composition gradually shifted. The total clonal abundance of ANMEs in the archaeal 16S rRNA gene and 16S rRNA clone libraries gradually increased, and phylotypes representing the three ANME groups (i.e., ANME-1, -2 and -3) were identified, although ANME-1 and -3 phylotypes were not detected in the inoculum sediment by the 16S rRNA gene clone analysis. The archaeal phylotype MK0D_A9, affiliated with ANME-2a, was the most abundant archaeal phylotype on day 2,013 (25.7% and 66.0% of the clones examined in the 2,013-day 16S rRNA gene and 16S rRNA libraries, respectively).

Interestingly, the methylotrophic methanogen genus *Methanococcoides* phylotype MK903D_A2 was predominantly detected in the 903-day 16S rRNA library, although no potential methylotrophic substrates for *Methanococcoides*, such as methanol or methylamines [50], were supplied in the bioreactor. Conceivably, the methylotrophic substrates were eluted as secondary products from the inoculum sediment [51]. Indeed, methylotrophic methanogens also grew in a previously reported DHS bioreactor for the cultivation of a subseafloor methanogenic community, although no methylotrophic substrate was provided to the bioreactor [42]. These methanotrophic/methanogenic archaeal components were also confirmed by methyl-coenzyme M reductase alpha-subunit gene (*mcrA*; a key gene in methane-oxidation/production) sequence-based clone analysis of the 2,013-day sample, and the results were generally consistent with those of the archaeal 16S rRNA gene sequence-based clone analysis (Fig. S6). One remarkable inconsistency was noted between the *mcrA* gene and 16S rRNA gene sequence-based clone libraries: phylotypes related to *Methanococcoides* were the dominant component in the *mcrA* mRNA-based library (65.3% of the clones examined), whereas the ANME-2a phylotype was the dominant component in the 2,013-day archaeal 16S rRNA gene and 16S rRNA libraries. This inconsistency may have been caused by the biases associated with PCR amplification and cloning, but the potential involvement of *Methanococcoides* members in AOM could not be excluded.

In addition to the methanotrophic/methanogenic archaeal phylotypes, many phylotypes related to other diverse uncultured archaeal lineages were also identified in the bioreactor enrichment. The 16S rRNA gene and 16S rRNA phylotypes related to DSAG and MBG-D were relatively abundant, comprising 10.3–25.6% and 9.3–21.9% of the archaeal 16S rRNA gene sequence-based clone libraries, respectively. Other uncultured archaeal lineages, such as MCG, Miscellaneous Euryarchaeotic Group (MEG), Deep-Sea Euryarchaeotic Group-2 (DSEG-2), Marine Hydrothermal Vent Group (MHVG), and Ancient Archaeal Group (AAG), were detected as minor components following the long-term incubation.

The dominant bacterial components in the inoculum sediment (i.e., the 0-day sample in Fig. 3B) were related to members of the *Deltaproteobacteria*, *Gammaproteobacteria*, and *Chloroflexi*, which have been frequently detected as dominant bacterial components in subseafloor sediments, including methane seeps and mud volcanoes [52]–[55]. Putative sulfate-reducing deltaproteobacterial partners of ANMEs (SEEP-SRB1, SEEP-SRB2, *Desulfobacteraceae*, and *Desulfobulbaceae*; Fig. S5B) were detected in the inoculum sediment (30.0% of the clones examined). During incubation in the DHS bioreactor, the bacterial community gradually shifted. The putative SRB partners were the dominant bacterial populations until day 903, and then gammaproteobacterial phylotypes became the dominant population. The bacterial phylotype MK903D_B5, which is affiliated with an uncultured gammaproteobacterial lineage, was the dominant bacterial phylotype on day 2,013 (Fig. S5C; 20.0% and 50.4% of the clones examined in the 2,013-day 16S rRNA gene and 16S rRNA clone libraries, respectively). This gammaproteobacterial phylotype was not detected in the inoculum sediment, but its population gradually increased during the bioreactor operation period. The second most abundant bacterial phylotype on day 2,013, MK903D_B19, is also affiliated with the *Gammaproteobacteria* and is closely related to the aerobic methanotrophic genus *Methylobacter* (Fig. S5C; 16.0% and 32.2% of the clones examined in the 2,013-day 16S rRNA gene and 16S rRNA clone libraries, respectively). Other gammaproteobacterial aerobic methanotrophic genera, such as *Methylomonas* and *Methylophaga*, were also detected in small numbers (Fig. S5C). The occurrence of these aerobic methanotrophs indicated the contamination of a small amount of molecular oxygen in the bioreactor. In fact, some minor bacterial phylotypes (MK903D_B6, MK903D_B31, MK903D_B42, MK903R_B71, MK903R_B75, MK1732D_B15, MK1732D_B55, and MK2013D_B26) were shown to be closely related to aerobic bacterial isolates (≥97.0% 16S rRNA gene sequence identity in the National Center for Biotechnology Information 16S Microbial database; data not shown). In addition to the delta- and gamma-proteobacterial phylotypes, other phylogenetically diverse bacterial phylotypes were detected following incubation, and the overall bacterial diversity spanned 24 bacterial phyla/lineages (Fig. S5).

As described above, our 16S rRNA gene and *mcrA* gene sequence-based clone analyses demonstrated that the microbial community shifted during operation of the bioreactor, and phylogenetically diverse yet-to-be-cultured microorganisms were successfully cultivated in the DHS bioreactor. The changes in the composition of the microbial community were further confirmed by archaeal and bacterial 16S rRNA gene-based T-RFLP analysis of bioreactor incubation samples collected at days 285, 1,376, 1,529, in addition to the samples used for clone analysis, as mentioned above (Fig. S7 and S8). The detection of unassigned T-RFs indicated the presence of microbial components that could not be identified by the 16S rRNA gene sequence-based clone analysis.

### Microbial diversity and richness in the DHS bioreactor incubation samples

To evaluate the diversity and richness of the DHS bioreactor incubation samples, Chao1 species richness, Shannon diversity index, Evenness, clone library coverage, and rarefaction curves were calculated for all clone libraries (Table 1 and Fig. S9). The rarefaction curves for the bioreactor incubation samples, except the *mcrA* mRNA-based clone library, did not plateau (Fig. S9), and the coverage values did not reach 100% in any of the clone libraries (51–99%; Table 1). These results indicated that additional archaeal and bacterial phylotypes would be found in the incubation samples with additional sequencing. Interestingly, the Chao1 species richness, Shannon diversity index, and rarefaction curves for the archaeal 16S rRNA gene clone libraries indicated that the archaeal diversity of the 2,013-day incubation sample was higher than that of the inoculum sediment (Table 1 and Fig. S9A). These results suggest that the abundance of minor archaeal components, which could not be detected in the inoculum sediment, increased in the bioreactor. The existence of minor and diverse archaeal components in the same sediment core sample has been revealed by 16S rRNA gene tag-sequencing analysis [30]. In contrast to the results obtained from analysis of the archaeal clone libraries, statistical analyses of the bacterial clone libraries suggested that the bacterial diversity gradually decreased during the bioreactor operation period (Table 1 and Fig. S9B).

**Table 1:** Statistical analysis of clone libraries.

Clone library	Total clone number	Phylotype^a^ number	Chao1 species richness^b^	Shannon diversity index	Evenness	Coverage (%)
Archaeal 16S rRNA gene						
0-day DNA	93	22	26 (23–41)	2.71	0.88	92
903-day DNA	117	26	42 (30–84)	2.58	0.79	89
903-day RNA	86	23	45 (29–110)	2.55	0.81	86
1,732-day DNA	117	37	83 (53–169)	2.63	0.73	79
2,013-day DNA	105	31	58 (39–121)	2.76	0.80	84
2,013-day RNA	106	13	24 (15–67)	1.34	0.52	93
Bacterial 16S rRNA gene						
0-day DNA	90	62	135 (95–223)	3.98	0.96	51
903-day DNA	93	45	99 (65–190)	3.49	0.92	70
903-day RNA	89	54	115 (80–197)	3.76	0.94	58
1,732-day DNA	153	53	89 (68–143)	3.33	0.84	80
2,013-day DNA	100	35	56 (42–97)	2.92	0.82	80
2,013-day RNA	115	15	27 (18–69)	1.43	0.53	92
*mcrA* gene						
2,013-day DNA	80	12	15 (13–34)	1.78	0.72	94
2,013-day RNA	121	6	6 (6–6)	1.14	0.64	99

a A phylotype was defined as ≥97% sequence identity.

b Numbers in parentheses indicate the 95% confidence interval.

### Detection of microbial cells in the DHS bioreactor using FISH

**Figure 4:**
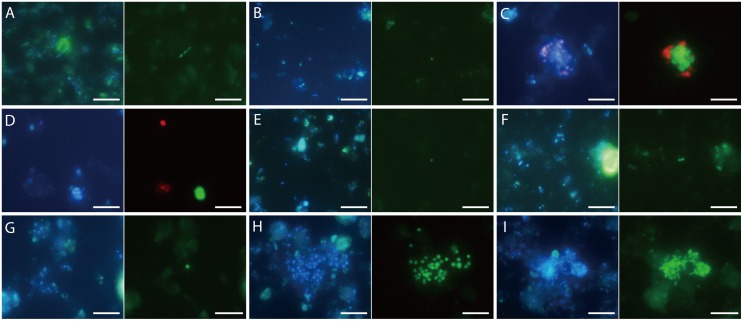
(A and G–I) FISH and (B–F) CARD-FISH images of microbial cells cultivated in the DHS bioreactor. Photomicrographs of DAPI-stained cells (left) and epifluorescence (right) showing identical fields. (A) Chain-forming ANME-1-350-stained cells in the 2,013-day sample (Alexa Fluor 488, green). (B) An ANME-2a-647-stained coccoid-shaped cell in the 2,013-day sample (Fluorescein, green). (C and D) Color overlay of ANME-2c-760- (Fluorescein, green) and EUB338-stained cells (Alexa Fluor 594, red) in the 903-day sample. (E) A MBGB-380-stained coccoid-shaped cell in the 2,013-day sample (Fluorescein, green). (F) A MBGD-318-stained rod-shaped cell in the 2,013-day sample (Fluorescein, green). (G) A MCOCID442-stained coccoid-shaped cell in the 2,013-day sample. (H) Mγ669-stained coccoid-shaped cells in the 2,013-day sample (Alexa Fluor 488, green). (I) UncGAM731-stained irregular rod-shaped cells in the 2,013-day sample (Alexa Fluor 488, green). Bars represent 10 µm.

The presence of active microorganisms in the DHS bioreactor was further confirmed by standard FISH and CARD-FISH analyses of the 903- and 2,013-day samples (Fig. 4 and Table S3). The ANME-1-350 probe-stained cells had a typical ANME-1-like rectangular morphology and formed chains of two or three cells (Fig. 4A). All ANME-2a cells visualized with the ANME-2a-647 probe occurred as single cells without any bacterial partners (Fig. 4B). The cells of ANME-2a were small cocci of approximately 0.4–0.6 µm in diameter. The ANME-2c cells stained with the ANME-2c-760 probe in the 903-day sample formed aggregates with bacterial partners, which were stained with the EUB338 probe (Fig. 4C), or occurred as aggregates without bacterial cells (Fig. 4D). Most of the ANME-2c aggregates detected did not occur with bacterial cells (11 of 15 aggregates examined in this experiment). In the 2,013-day sample, no signals of the ANME-2c-760 probe were identified in the triplicate CARD-FISH experiments. All of the MBGB-380-stained cells were small coccoid-shaped cells with a size of approximately 0.4–0.6 µm (Fig. 4E), and the cell morphology and size were consistent with those of DSAG archaeal cells detected in Black Sea microbial mats [8]. The MBG-D archaeal cells detected by the MBGD-318 probe were straight rods (approximately 2–3 µm long and 0.5–1 µm wide) with blunt ends and a sheath-like structure (Fig. 4F). Almost all of the cells stained by the Mγ669 probe morphologically resembled *Methylobacter* species (Fig. 4H) and formed cysts that are normally found under conditions of oxygen deprivation and desiccation [56]. The UncGAM731 probe-positive cells were irregular rod-shaped cells (Fig. 4I; approximately 1–2 µm long and 0.5–1 µm wide). These uncultured gammaproteobacterial cells formed monospecies aggregates (Fig. 4I) and did not form aggregates with ANME cells.

## Discussion

We used a continuous-flow DHS bioreactor system to cultivate an AOM microbial community from deep-sea methane-seep sediment collected from the Nankai Trough, Japan. In this study, 24.7 mM sulfate was fed as the dominant electron acceptor for the AOM microbial community. The potential AOM activity in the DHS bioreactor is in the range of seep and mud volcano samples [2]. However, it was probably low compared to what could be achieved in high-pressure bioreactors [18], [21] (Table 2). Due to the extremely low energy yield of the net AOM reaction (ΔG°′ = −16 kJ mol^−1^ in the case of sulfate-dependent AOM [2]), the reaction is strongly influenced by substrate and product concentrations. In terms of the poor solubility of methane in solution at ambient pressure (∼2 mM CH_4_ at 10°C [57]), high pressure-type cultivation systems have the advantages of *in vitro* stimulation of AOM [18], [21], [58]. In other words, effective methane supply is thought to be an important factor for the cultivation of AOM microbial communities when using ambient-pressure type bioreactor systems. Meulepas *et al*. [20] used an ambient-pressure submerged-membrane bioreactor system and achieved significantly higher potential AOM activity (286 µmol g-dw^−1^ day^−1^) after 884 days of operation. In the submerged-membrane bioreactor system, the AOM microbial community was continuously sparged by methane gas bubbles for an ideal methane supply, but the gas-sparging method may expose microorganisms to relatively high shear forces. In contrast, the DHS bioreactor has a good gas-exchange capability because of a relatively large gas-liquid interface and a relatively short substrate transportation distance between the gas-liquid interface and the center of sponges. This unique feature of the DHS bioreactor may have enabled the successful long-term cultivation of the AOM microbial community under a non-turbulent ambient methane pressure condition.

**Table 2:** Comparison of AOM enrichments in different types of continuous-flow bioreactors.

Incubation technique name	CH_4_ pressure	Influent sulfate concentration	Incubation temperature	Sediment (water depth)	Maximum AOM rate	Involved ANMEs^d^	Reference
Down-flow hanging sponge (DHS) bioreactor	Ambient	25 mM	10°C	Nankai Trough (2,533 m)	375 nmol g-dw^−1^ day^−1^	ANME-1, **ANME-2a**, ANME-2c, and ANME-3	This study
Anaerobic sediment incubator system (AMIS)	Ambient	28 mM^b^	5°C	Monterey Bay (962 m)^b^	138 nmol g-dw^−1^ day^−1b^	**ANME-1** and ANME-2c^b^	[19], [25]
Submerged membrane bioreactor	Ambient	21 mM	15°C	Eckernförde Bay (28 m)	286 µmol g-dw^−1^ day^−1^	**ANME-2a**	[20], [65]
Flow-through system	Ambient^a^	28 mM^a^	4−6°C	Black Sea (326 m)	0.65 µmol g-dw^−1^ day^−1a^	NR	[22]
				Hydrate Ridge (776 m)	0.55 µmol g-dw^−1^ day^−1a^	NR	
				Gullfaks (150 m)	0.12 µmol g-dw^−1^ day^−1a^	NR	
Sediment flow-through (SLOT) system	Ambient	19 mM^c^	10°C	Eckernförde Bay (28 m)	5.32 nmol cm^−3^ day^−1c^	NR	[23]
Continuous high-pressure bioreactor	8 MPa	9 mM	15°C	Gulf of Cadiz (1,200 m)	9.22 µmol g-dw^−1^ day^−1^	**ANME-2a** and ANME-3	[21], [26]
High-pressure continuous incubation system (HP-CI system)	10 MPa	8 mM	20°C	Black Sea (213 m)	0.37 mmol g-dw^−1^ day^−1^	NR	[18]

NR: not reported.

a Data from long-term incubations with short columns described in Wegener and Boetius [22].

b Data from high-flow experiments with NON-SEEP sediments described in Girguis *et al*. [25].

c Data from new high flow core experiments described in Steeb *et al*. [23].

d Dominant ANME type is shown in bold.

Interestingly, the 16S rRNA gene-based quantitative real-time PCR data showed that the archaeal and bacterial populations in the DHS bioreactor responded differently to the incubation. The bacterial 16S rRNA gene copy number increased approximately tenfold during the period of incubation, whereas the archaeal 16S rRNA gene copy numbers remained roughly the same throughout the 2,013 days of incubation (Fig. 2). Additionally, statistical analyses of the 16S rRNA gene clone libraries showed that archaeal diversity after 2,013 days of incubation was higher than in the inoculum sediment, whereas the bacterial diversity decreased during the period of bioreactor operation (Table 1 and Fig. S9). These different behaviors may have resulted from differences in survival mechanisms between the archaeal and bacterial communities, such as membrane structure and energy conversion efficiency [59].

Our 16S rRNA gene sequence-based molecular analyses suggest that ANME-2a is the dominant anaerobic methane oxidizer in the DHS bioreactor. In contrast, the relative abundance of ANME-2c-related clones decreased during the period of bioreactor operation, and ANME-2c cells could not be detected by CARD-FISH in the 2,013-day sample. ANME-2a and -2c are both categorized as members of ANME-2 [8], but ANME-2a and -2c are phylogenetically distinct at the genus and family levels (e.g., the predominant ANME-2a phylotype MK0D_A9 has 89.8% 16S rRNA gene sequence identity to the ANME-2c phylotype MK0D_A1). Thus, ANME-2a and -2c may differ in their physiological properties, and the physical-chemical conditions in the DHS bioreactor cultivation appear to favor enrichment of ANME-2a. The physical-chemical conditions potentially controlling the occurrence of specific ANME groups are still under debate [8], [25], [60]–[62], but the environmental distributions of intact polar membrane lipids, and *mcrA* gene and 16S rRNA gene sequences have indicated that ANME-2 dominates in relatively sulfate-rich or high-sulfate-flux environments [61]–[64]. The dominance of ANME-2a populations has also been confirmed in a submerged-membrane bioreactor system fed with high concentrations of sulfate (Table 2) [20], [65]. In addition to the sulfate concentration, the redox potential is thought to be a significant variable affecting the occurrence of ANME-2a. The habitats typically dominated by ANME-2 and -3 members are characterized by higher dissolved oxygen (DO) contents (i.e., higher redox environments) than ANME-1-dominated habitats [8], [61]. A high-pressure continuous-flow AOM bioreactor was also reported to be dominated by ANME-2a and gammaproteobacterial aerobic methanotroph populations [21], [26]. Therefore, assuming that contamination with a small amount of molecular oxygen is inevitable in continuous-flow bioreactor systems, the preferred enrichment of ANME-2a may be explained by the combination of high-sulfate and relatively more oxidative conditions. The effect of the incubation temperature (i.e., 10°C) on the predominance of ANME-2a is unlikely to be significant because some ANME-1 and -2 members are metabolically active at ∼10°C [60].

FISH and 16S rRNA clone analyses revealed that active ANME-1 cells also thrived in the DHS bioreactor. ANME-1 members are frequently observed in the deeper zones of subseafloor sediments and in the internal section of microbial mats [62]–[64], [66], indicating that members of ANME-1 prefer to inhabit highly reductive environments. Considering these previous observations and the possible occurrence of DO concentration gradients in the sponge carriers [67]–[68], the ANME-1 cells may colonize the interior portion of the sponge carriers and/or the lower portion of the DHS bioreactor, where redox potentials are comparatively low. In addition, the coexistence of three ANME groups (i.e., ANME-1, -2, and -3) in the DHS bioreactor is interesting because their co-occurrence has only been reported in some marine sediments [2], [64]. This co-occurrence may have resulted from physical-chemical gradients along bioreactor height and/or sponge carrier depth. It should be noted that the composition of the ANME groups in inoculum sediments would significantly affect the cultivation results, along with physical-chemical factors.

The presence of ANME-1, -2a, and most ANME-2c cells without close physical interaction with bacterial cells (Fig. 4) is intriguing because ANMEs may require a close physical interaction with their syntrophic SRB partners to perform AOM [69]–[71]. AOM without syntrophic SRB partners is still largely unknown, but a bacteria-independent AOM mechanism has been recently proposed by Milucka *et al*. [72]. In contrast, the involvement of SRB in AOM cannot be denied because some potential sulfate-reducing deltaproteobacterial phylotypes (i.e., *Desulfobacteraceae* and SEEP-SRB1) were detected, even after 2,013 days of enrichment (Fig. S5B). Further enrichment and multicomponent investigations may shed more light on the mechanisms of AOM.

In addition to ANMEs, other diverse uncultured archaeal lineages, such as DSAG, MBG-D, MEG, DSEG-2, MCG, MHVG, and AAG, were also identified in the DHS bioreactor incubation samples. DSAG members are known to be a predominant archaeal component of subseafloor sediments [73]–[75]. Metabolically active DSAG members have frequently been detected within sulfate-methane transition zones, where AOM is prominent [9], [11]. A previous study of the stable carbon isotopic composition of archaeal lipids/cells suggested that DSAG might assimilate sedimentary organic matter as a carbon source while performing AOM for energy generation [9]. The occurrence of active DSAG phylotypes in our bioreactor provides further evidence that DSAG may be sustained by AOM. However, current studies based on the co-variation of DASG archaeal 16S rRNA gene abundance and geochemical data suggest that members of DSAG are heterotrophic and involved in the iron and/or manganese cycle [76]–[77]. In our bioreactor, we supplied iron and manganese compounds as trace elements. DHS bioreactor systems are also known to possess the ability to maintain a relatively large biomass [29]. Therefore, the presence of iron, manganese, and a higher concentration of biomass-derived organic compounds may enhance the growth of DSAG phylotypes. MBG-D, a *Thermoplasmata*-related uncultured archaeal lineage, is globally distributed in marine sediments [78] and has been co-detected with ANME-2a in some AOM enrichment cultures [19], [26], [65]. Recent single-cell genomic sequencing has revealed that members of MBG-D are likely anaerobic protein-degrading microorganisms [78]. Thus, active MBG-D phylotypes may utilize dead biomass produced in the bioreactor and/or extracellular organic matrix, which may be excreted by ANME populations [2], [66]. Other uncultured archaeal lineages, such as MEG, DSEG-2, MCG, MHVG, and AAG, may be metabolically less active in the DHS bioreactor because their sequences either were not retrieved or were less abundant in the 16S rRNA clone libraries (Fig. 3A, S3 and S4). In contrast, the detection of such previously uncultured archaeal lineages after long-term cultivation indicates that these archaeal lineages can be cultured under laboratory conditions mimicking methane-seep environments.

At the end of the long-term cultivation, gammaproteobacterial phylotypes were the dominant bacterial population. The dominant gammaproteobacterial phylotype, MK903D_B5, is phylogenetically similar to a chemoautotrophic sulfur-oxidizing endosymbiont group (e.g., 91.7% 16S rRNA gene sequence identity to an endosymbiont of *Alviniconcha* sp.; Fig. S5C). However, the physiological characteristics of this phylotype remain largely unknown because no closely related isolate exists. Thus, further analyses are needed to clarify the ecological roles of this phylotype. Detection of the *Methylococcales*, *Methylophaga*, and other aerobic bacterial phylotypes indicates that molecular oxygen may have contaminated the medium through the Viton tubing before entering the PVC column. However, the reducing condition of the effluent medium indicates removal of the dissolved molecular oxygen. The dissolved oxygen was likely reduced by these bacterial lineages during retention of the medium in the sponge carriers. An alternative explanation for the significant increase in the *Methylococcales* phylotypes in the oxygen-limited DHS bioreactor is that they might gain energy by methane-based fermentation metabolism, as recently shown by Kalyuzhnaya *et al*. [79]. Among the other diverse bacterial lineages, WS3, BD1-5, and OD1 were relatively abundant phylum-level uncultured bacterial lineages in the incubation samples (Fig. 3B). These bacterial lineages have also been detected in marine sediments, including methane seeps and mud volcanoes [54]–[55], [62], [80], and metagenomic studies of acetate-amended aquifers revealed that members of OD1 and BD1-5 play an important role in molecular hydrogen production, sulfur cycling, and anaerobic fermentation of sedimentary carbon [81]–[82]. The biogeochemical roles of these bacterial lineages in marine environments are not yet fully understood, but these lineages probably contribute to the cycling of methane-derived carbon in methane-seep ecosystems.

Our results show that ANMEs and many phylogenetically diverse yet-to-be-cultured microorganisms in the Nankai Trough methane-seep sediment can be cultured using the DHS bioreactor system described here. Further multicomponent investigations (e.g., subsequent isolation, (meta)genomic sequencing, and stable isotope labeling experiments) using DHS bioreactor enrichment cultures will enable the physiological characterization of the cryptic microorganisms in methane-seep ecosystems.

## Supporting Information

Figure S1 **Time-course changes in (A) ORP and (B) pH values of DHS bioreactor effluent.**
(PDF)

Figure S2 **The traces of (A) the difference in δ^13^C values of dissolved inorganic carbon between the 1,529-day sample supplemented with ^13^C-labelled methane and that supplemented with non-labeled methane, and (B) δ^13^C values of dissolved inorganic carbon in the 1,529-day sample supplemented with non-labeled methane.** Values of duplicate experiments are shown.
(PDF)

Figure S3 **Phylogenetic tree showing the affiliations of** ***Euryarchaeota*** **-related 16S rRNA gene and 16S rRNA phylotypes obtained in this study.** The phylotypes obtained in this study are shown in red, bold type. The initial tree was constructed with sequences that were longer than 1,000 nucleotides, using the neighbor-joining method. Shorter sequences were subsequently inserted into the tree using the parsimony insertion tool in the ARB program. Three crenarchaeotal sequences (*Aeropyrum pernix* K1 [D83259], *Sulfolobus acidocaldarius* ATCC 33909 [D14876], and *Thermofilum pendens* DSM 2475 [X14835]) were used as the outgroups (not shown). The numbers in parentheses indicate the number of phylotypes in each clone library and their frequency in each library in the following order: 16S rRNA gene clone library at day 0 (the inoculum sample), 16S rRNA gene clone library at day 903, 16S rRNA clone library at day 903, 16S rRNA gene clone library at day 1,732, 16S rRNA gene clone library at day 2,013, and 16S rRNA clone library at day 2,013. The scale bar represents the estimated number of nucleotide changes per sequence position. The symbols at the nodes show the bootstrap values (only those >75% are indicated) obtained after 1,000 resamplings.
(PDF)

Figure S4 **Phylogenetic tree showing the phylogenetic affiliations of** ***Crenarchaeota, Thaumarchaeota*** **, and deeply branching archaea-related 16S rRNA gene and 16S rRNA phylotypes obtained in this study.** The tree was constructed in the same manner as the *Euryarchaeota*-related 16S rRNA gene and 16S rRNA phylotypes (Fig. S3). Three bacterial sequences (*Bacillus subtilis* subsp. *subtilis* NCIB 3610 [ABQL01000001], *Escherichia coli* ATCC 11775 [X80725], and *Aquifex pyrophilus* Kol5a [M83548]) were used as the outgroups (not shown). The scale bar represents the estimated number of nucleotide changes per sequence position. The bold and colored sequences, symbols at the nodes, and numbers in the parentheses indicate the same meanings as in Fig. S3.
(PDF)

Figure S5 **Phylogenetic tree showing the phylogenetic affiliations of bacterial 16S rRNA gene and 16S rRNA phylotypes obtained in this study.** The initial tree was constructed with sequences longer than 1,200 nucleotides, using the neighbor-joining method. Shorter sequences were subsequently inserted into the tree using the parsimony insertion tool in the ARB program. Three archaeal sequences (*Methanosarcina acetivorans* C2A [AE010299], *Thermococcus profundus* DT5432 [Z75233], and *Nitrosopumilus maritimus* SCM1 [CP000866]) were used as the outgroups (not shown). (A) A large bacterial tree including diverse bacterial groups. (B–G) Expanded bacterial phylogenetic trees for (B) *Deltaproteobacteria*, (C) *Alphaproteobacteria*, *Betaproteobacteria*, *Gammaproteobacteria*, and *Epsilonproteobacteria*, (D) *Verrucomicrobia*, *Lentisphaerae*, *Chlamidiae*, and *Planctomycetes*, (E) *Bacteroidetes* and *Chlorobi*, (F) *Firmicutes* and *Tenericutes*, and (G) *Chloroflexi*. The scale bars represent the estimated number of nucleotide changes per sequence position. The bold and colored sequences, symbols at the nodes, and numbers in the parentheses indicate the same meanings as in Fig. S3.
(PDF)

Figure S6 **Phylogenetic tree showing the phylogenetic affiliations of deduced McrA amino acid sequences obtained in this study.** The numbers in parentheses indicate the number of phylotypes in each clone library and their frequency in each library in the following order: *mcrA* gene-based clone library at day 2,013, and *mcrA* mRNA-based clone library at day 2,013. The scale bar indicates 10% estimated sequence divergence. The meanings of the bold and colored sequences, and symbols at the nodes are the same as in Fig. S3.
(PDF)

Figure S7 **T-RFLP profiles of archaeal 16S rRNA genes digested with (A) HaeIII or (B) HhaI.** The phylogenetic affiliations of each T-RF were identified using the archaeal 16S rRNA gene and 16S rRNA clone sequences obtained in this study. The abbreviations for some peaks are as follows: Mcc, *Methanococcoides*; Msr, uncultured *Methanosarcinaceae*; UE, unclassified *Euryarchaeota*; and UA, unclassified *Archaea*.
(PDF)

Figure S8 **T-RFLP profiles of bacterial 16S rRNA genes digested with (A) HaeIII or (B) HhaI.** The phylogenetic affiliations of each T-RF were identified using the bacterial 16S rRNA gene and 16S rRNA clone sequences obtained in this study. The abbreviations for some peaks are as follows: ALF, *Alphaproteobacteria*; BET, *Betaproteobacteria*; GAM, *Gammaproteobacteria*; DEL, *Deltaproteobacteria*; EPS, *Epsilonproteobacteria*; and UB, unclassified *Bacteria*.
(PDF)

Figure S9 **Rarefaction curves for (A) archaeal and (B) bacterial 16S rRNA genes and 16S rRNA, and (C)** ***mcrA*** **genes and mRNA.**
(PDF)

Table S1 Oligonucleotide primers used in this study.
(PDF)

Table S2 16S rRNA-targeted oligonucleotide probes used in this study.
(PDF)

Table S3 Summary of FISH and CARD-FISH results.
(PDF)
