# Supplementary figures and images for: A Long-Term Cultivation of an Anaerobic Methane-Oxidizing Microbial Community from Deep-Sea Methane-Seep Sediment Using a Continuous-Flow Bioreactor
Source: PLoS One. 2014 Aug 20;9(8):e105356. doi: 10.1371/journal.pone.0105356 (PMC4139340; doi:10.1371/journal.pone.0105356)

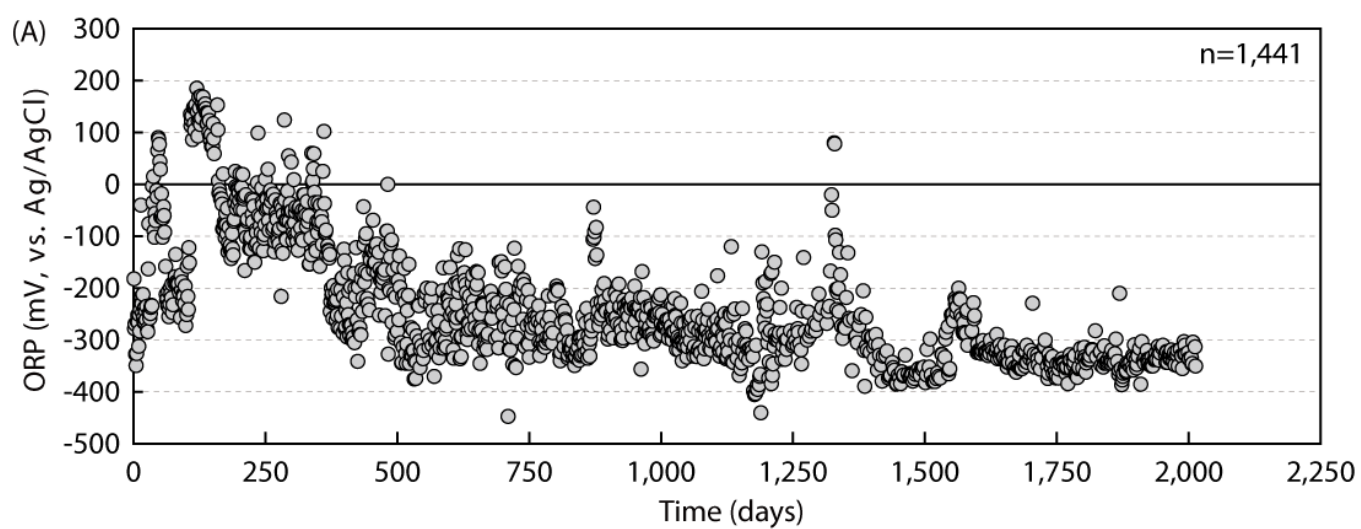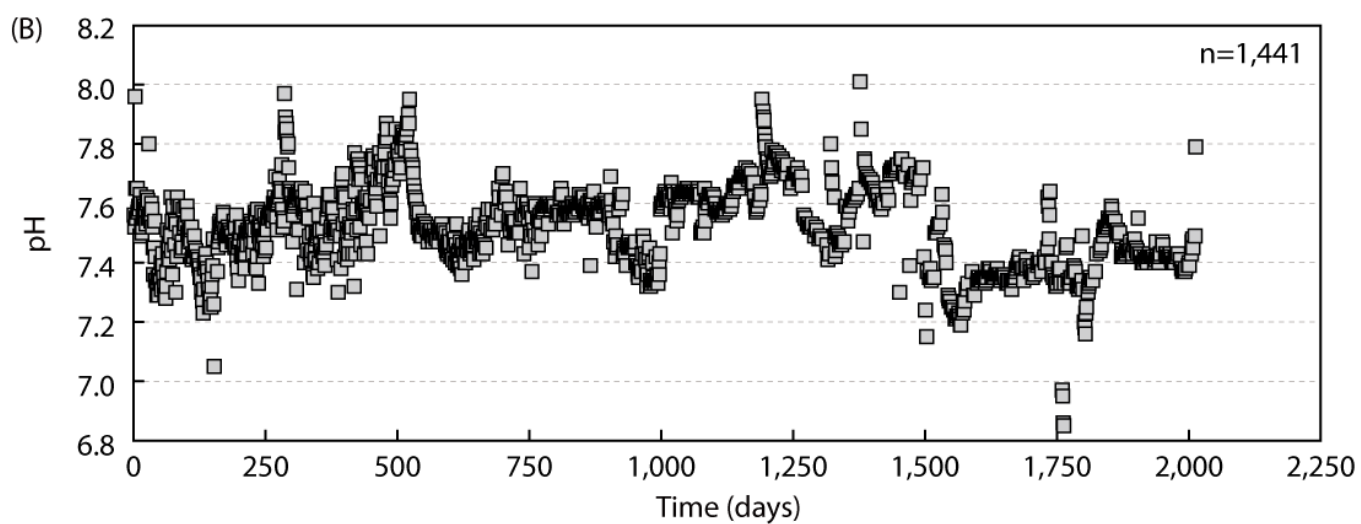

**Figure S1.** Aoki *et al.*

Supplement: Figure S1 — Time-course changes in (A) ORP and (B) pH values of DHS bioreactor effluent. (PDF) [file pone.0105356.s001.pdf]

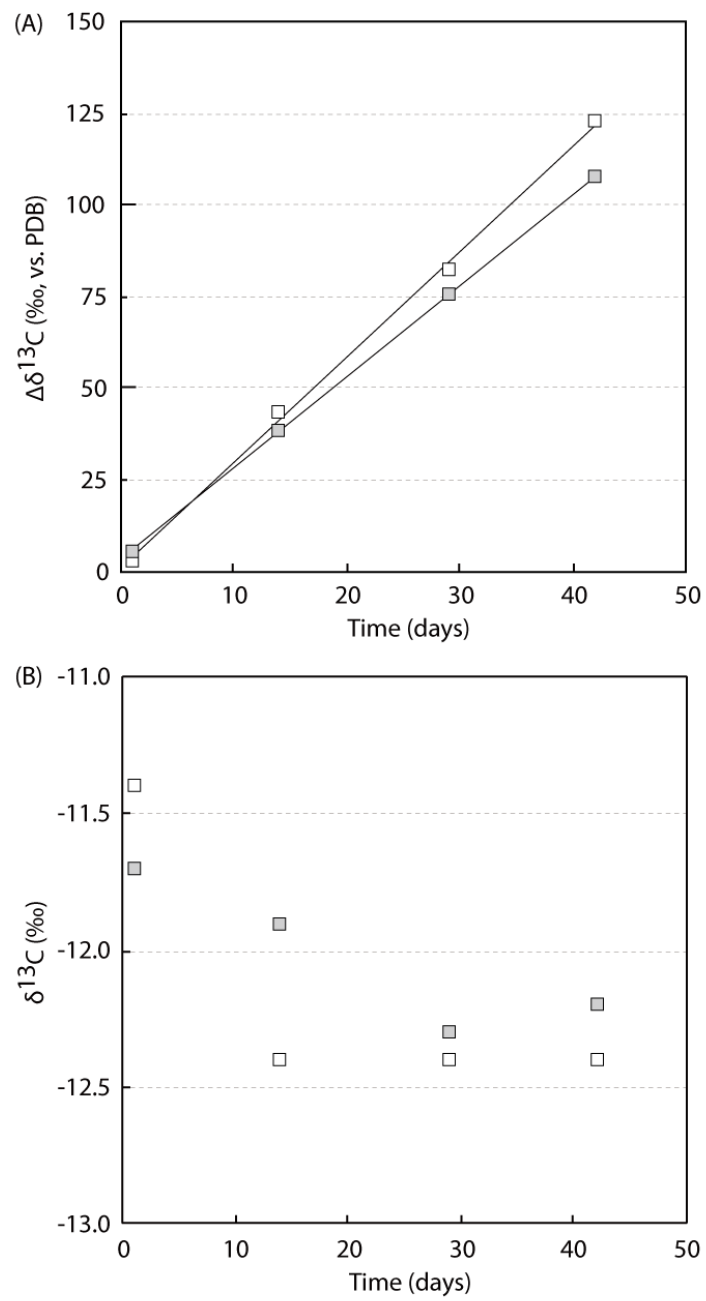

**Figure S2.** Aoki *et al.*

Supplement: Figure S2 — The traces of (A) the difference in δ13C values of dissolved inorganic carbon between the 1,529-day sample supplemented with 13C-labelled methane and that supplemented with non-labeled methane, and (B) δ13C values of dissolved inorganic carbon in the 1,529-day sample supplemented with non-labeled methane. Values of duplicate experiments are shown. (PDF) [file pone.0105356.s002.pdf]

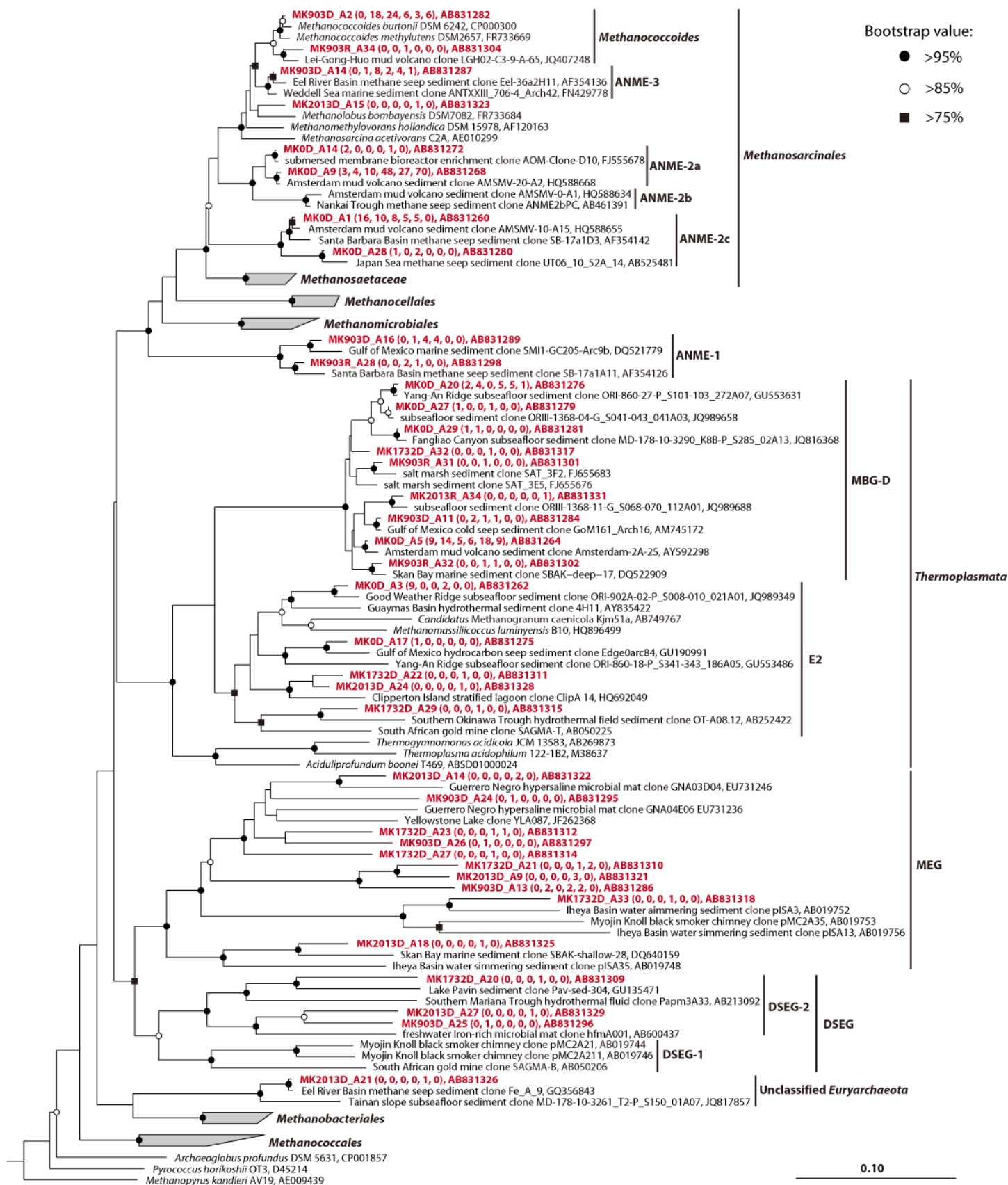

Figure S3 Aoki *et al.*

Supplement: Figure S3 — Phylogenetic tree showing the affiliations of Euryarchaeota -related 16S rRNA gene and 16S rRNA phylotypes obtained in this study. The phylotypes obtained in this study are shown in red, bold type. The initial tree was constructed with sequences that were longer than 1,000 nucleotides, using the neighbor-joining method. Shorter sequences were subsequently inserted into the tree using the parsimony insertion tool in the ARB program. Three crenarchaeotal sequences (Aeropyrum pernix K1 [D83259], Sulfolobus acidocaldarius ATCC 33909 [D14876], and Thermofilum pendens DSM 2475 [X14835]) were used as the outgroups (not shown). The numbers in parentheses indicate the number of phylotypes in each clone library and their frequency in each library in the following order: 16S rRNA gene clone library at day 0 (the inoculum sample), 16S rRNA gene clone library at day 903, 16S rRNA clone library at day 903, 16S rRNA gene clone library at day 1,732, 16S rRNA gene clone library at day 2,013, and 16S rRNA clone library at day 2,013. The scale bar represents the estimated number of nucleotide changes per sequence position. The symbols at the nodes show the bootstrap values (only those >75% are indicated) obtained after 1,000 resamplings. (PDF) [file pone.0105356.s003.pdf]

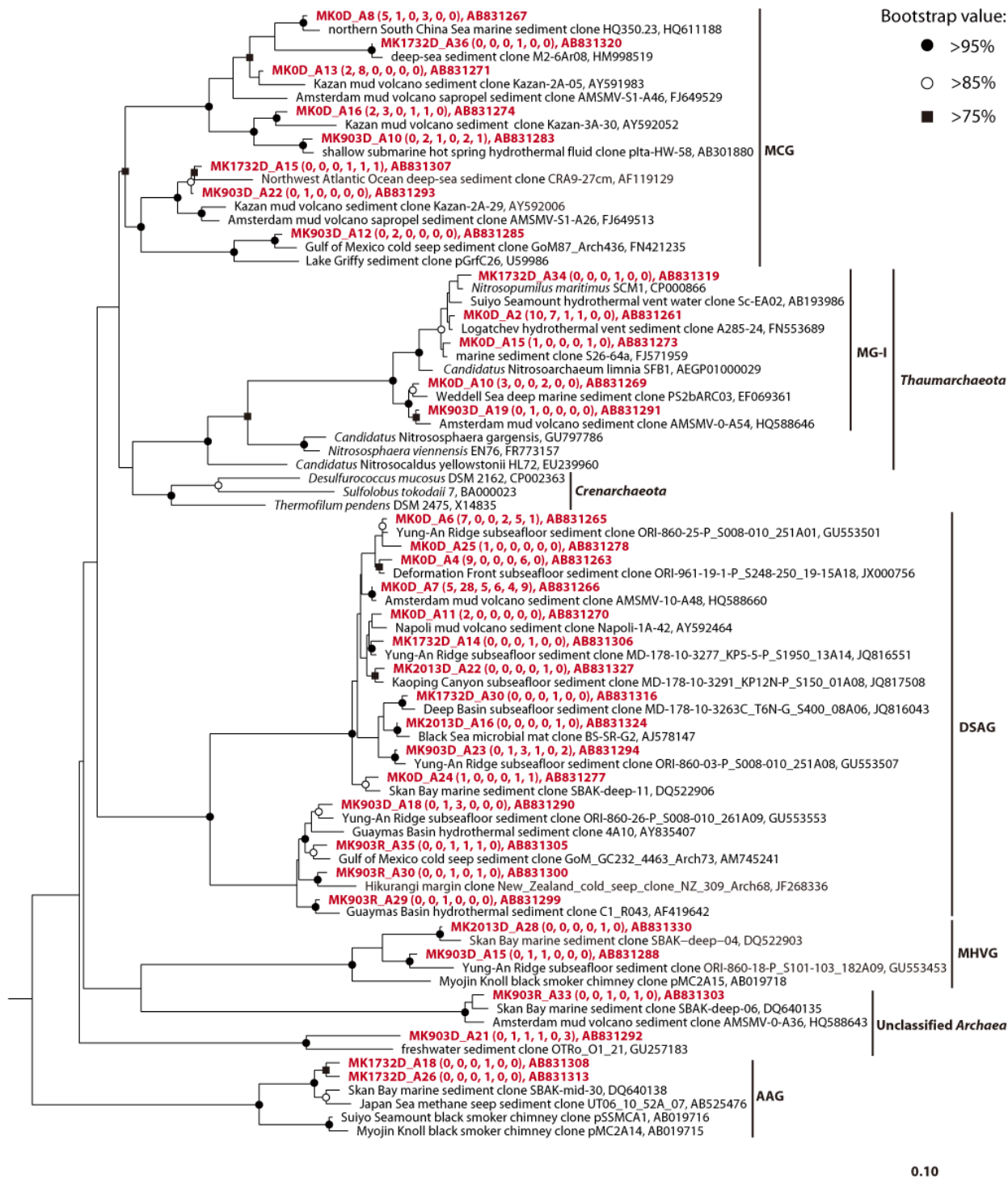

Figure S4. Aoki *et al.*

Supplement: Figure S4 — Phylogenetic tree showing the phylogenetic affiliations of Crenarchaeota, Thaumarchaeota , and deeply branching archaea-related 16S rRNA gene and 16S rRNA phylotypes obtained in this study. The tree was constructed in the same manner as the Euryarchaeota-related 16S rRNA gene and 16S rRNA phylotypes (Fig. S3). Three bacterial sequences (Bacillus subtilis subsp. subtilis NCIB 3610 [ABQL01000001], Escherichia coli ATCC 11775 [X80725], and Aquifex pyrophilus Kol5a [M83548]) were used as the outgroups (not shown). The scale bar represents the estimated number of nucleotide changes per sequence position. The bold and colored sequences, symbols at the nodes, and numbers in the parentheses indicate the same meanings as in Fig. S3. (PDF) [file pone.0105356.s004.pdf]

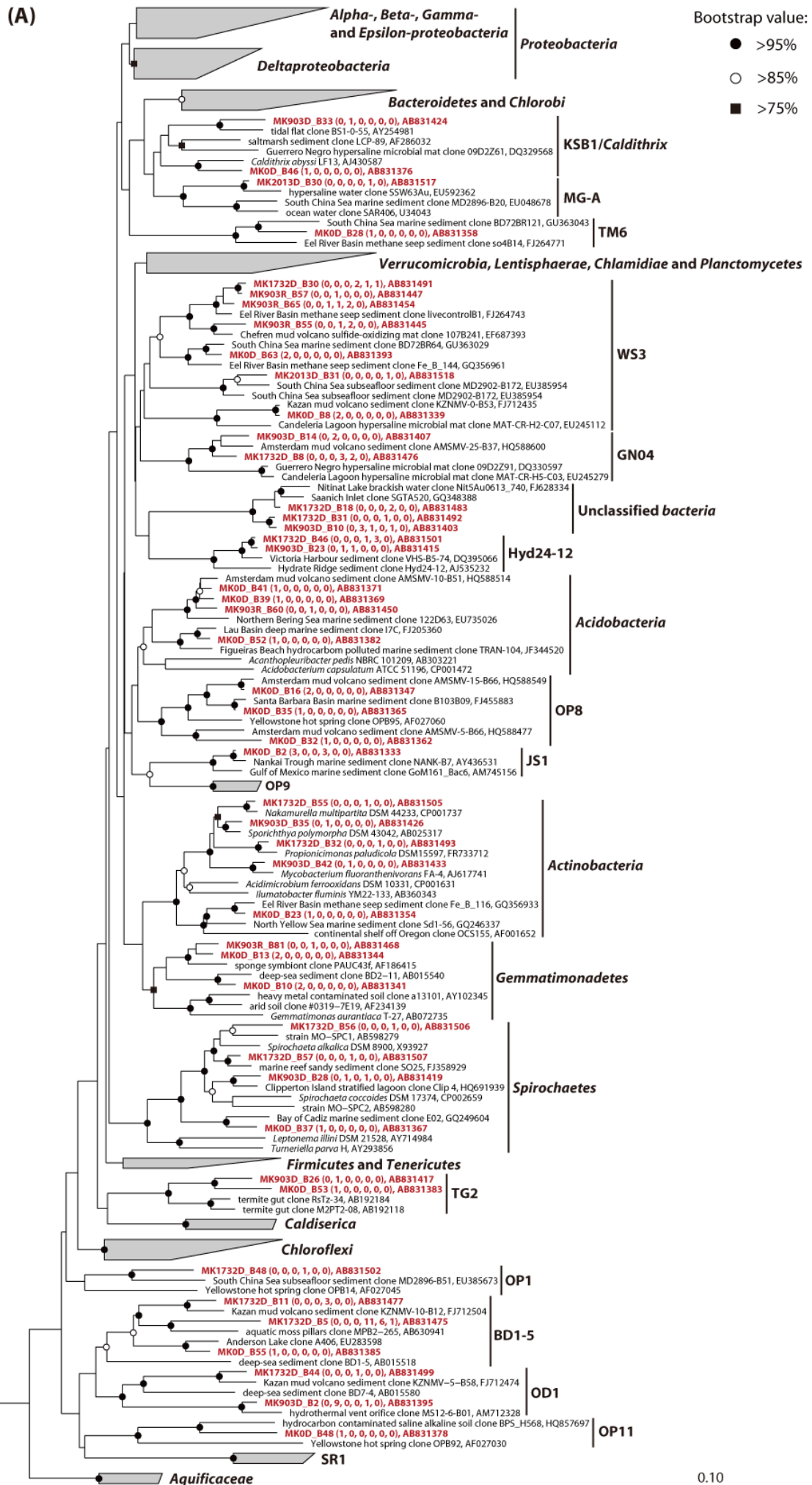

(B)

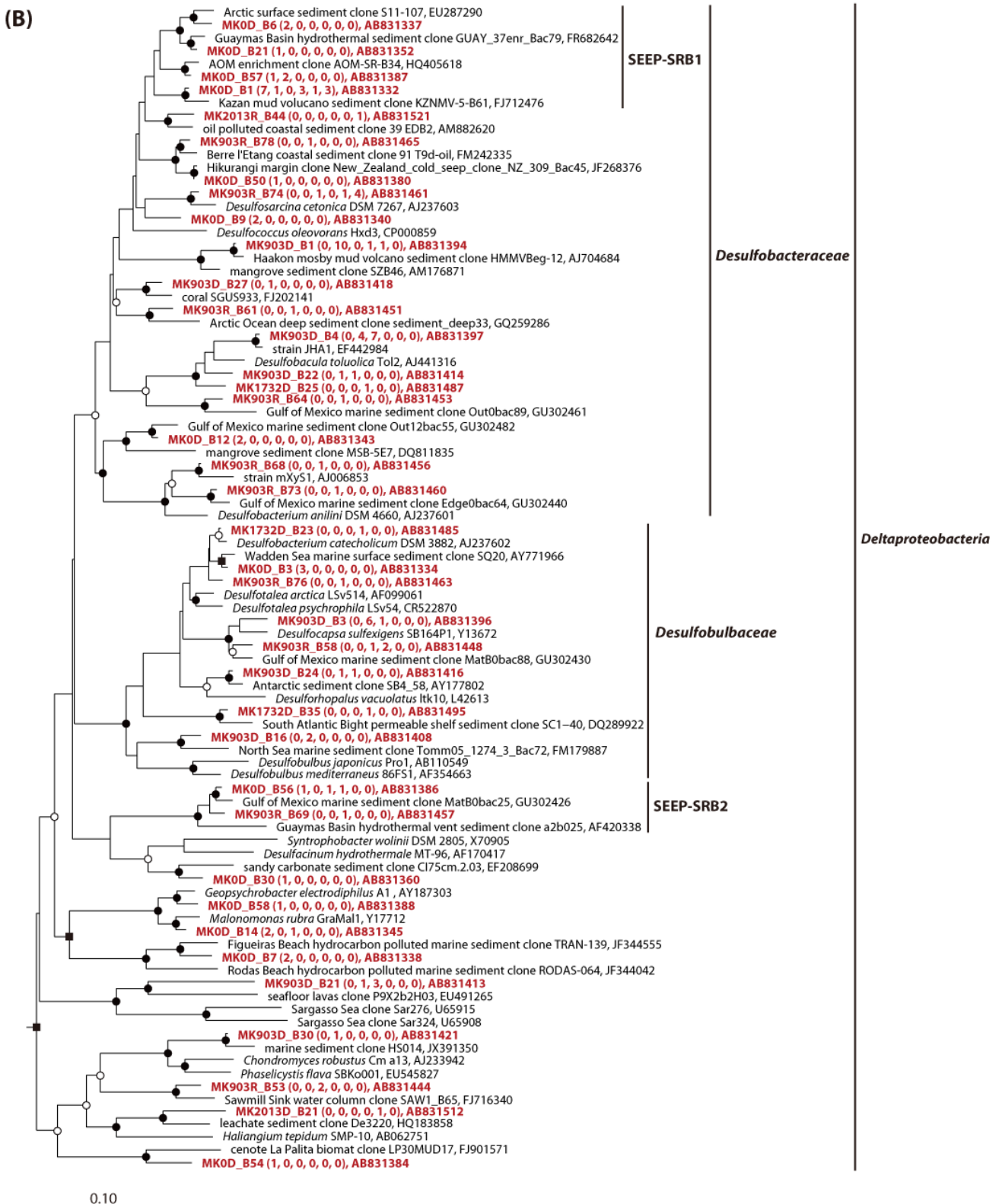

Figure S5. Aoki *et al.*

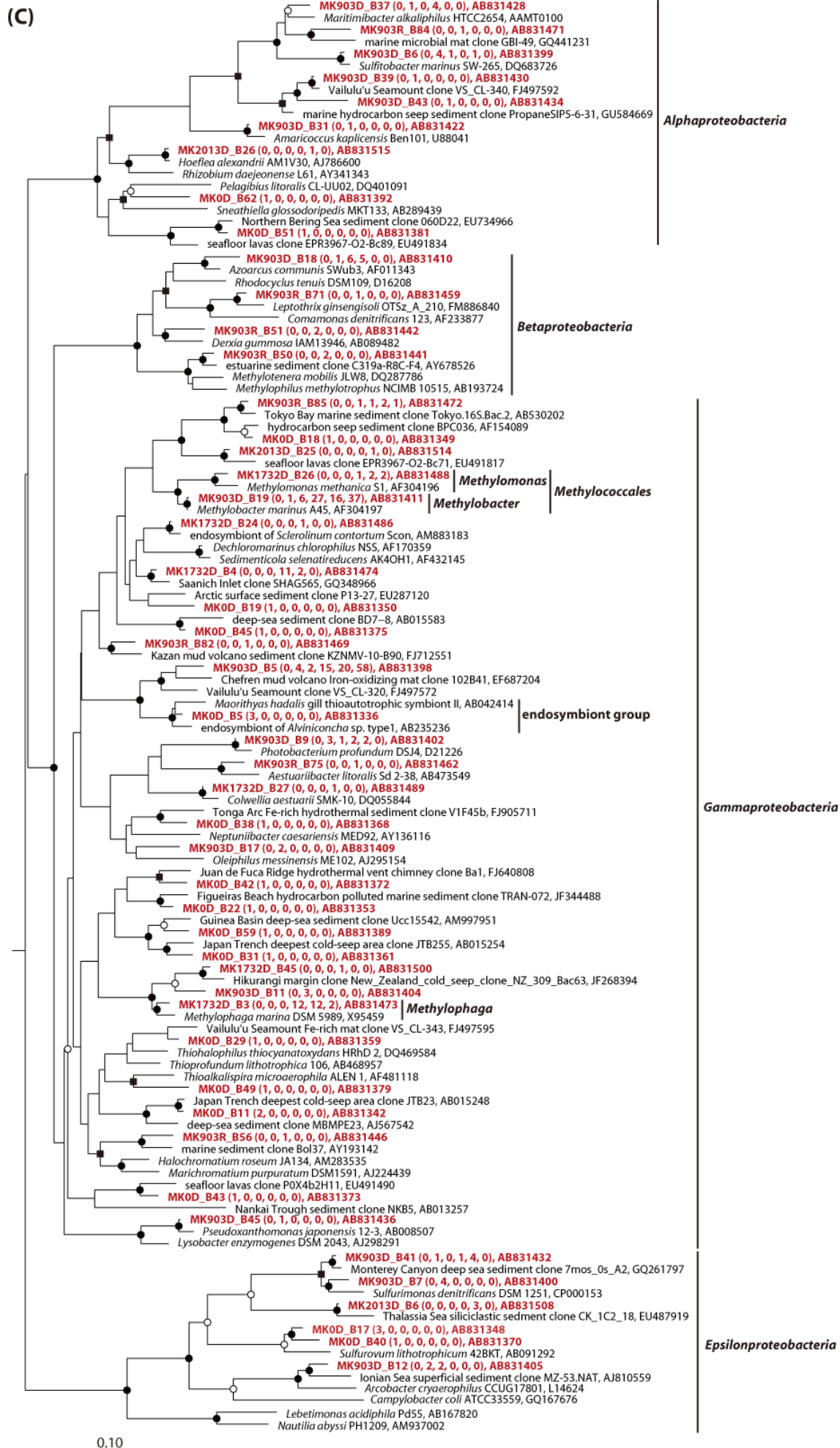

Figure S5. Aoki *et al.*

(D)

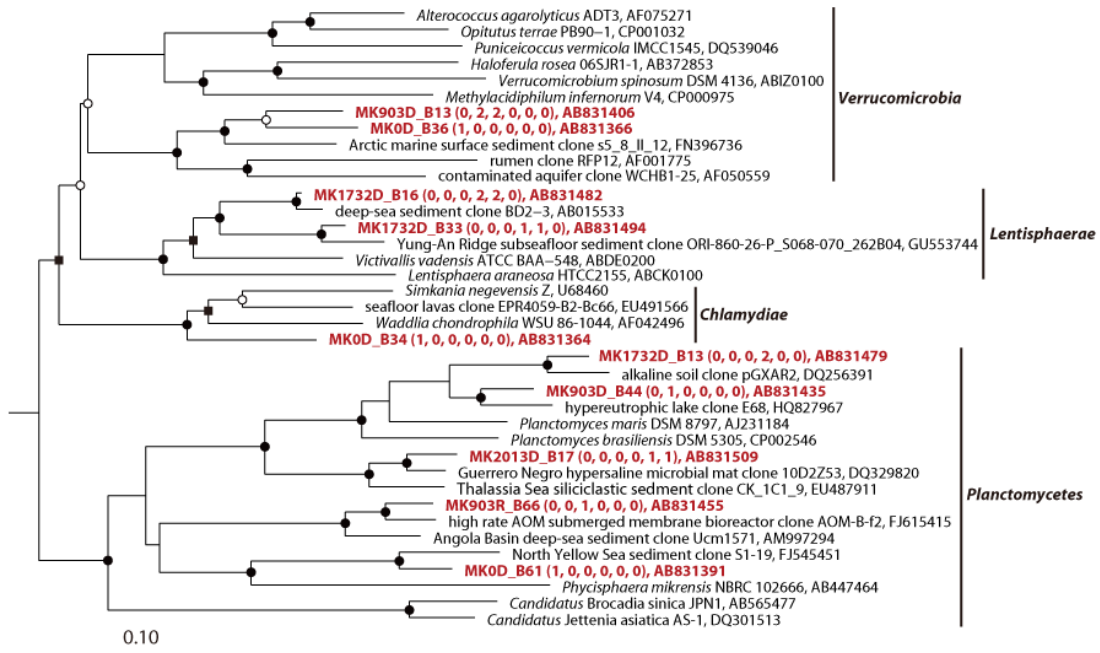

(E)

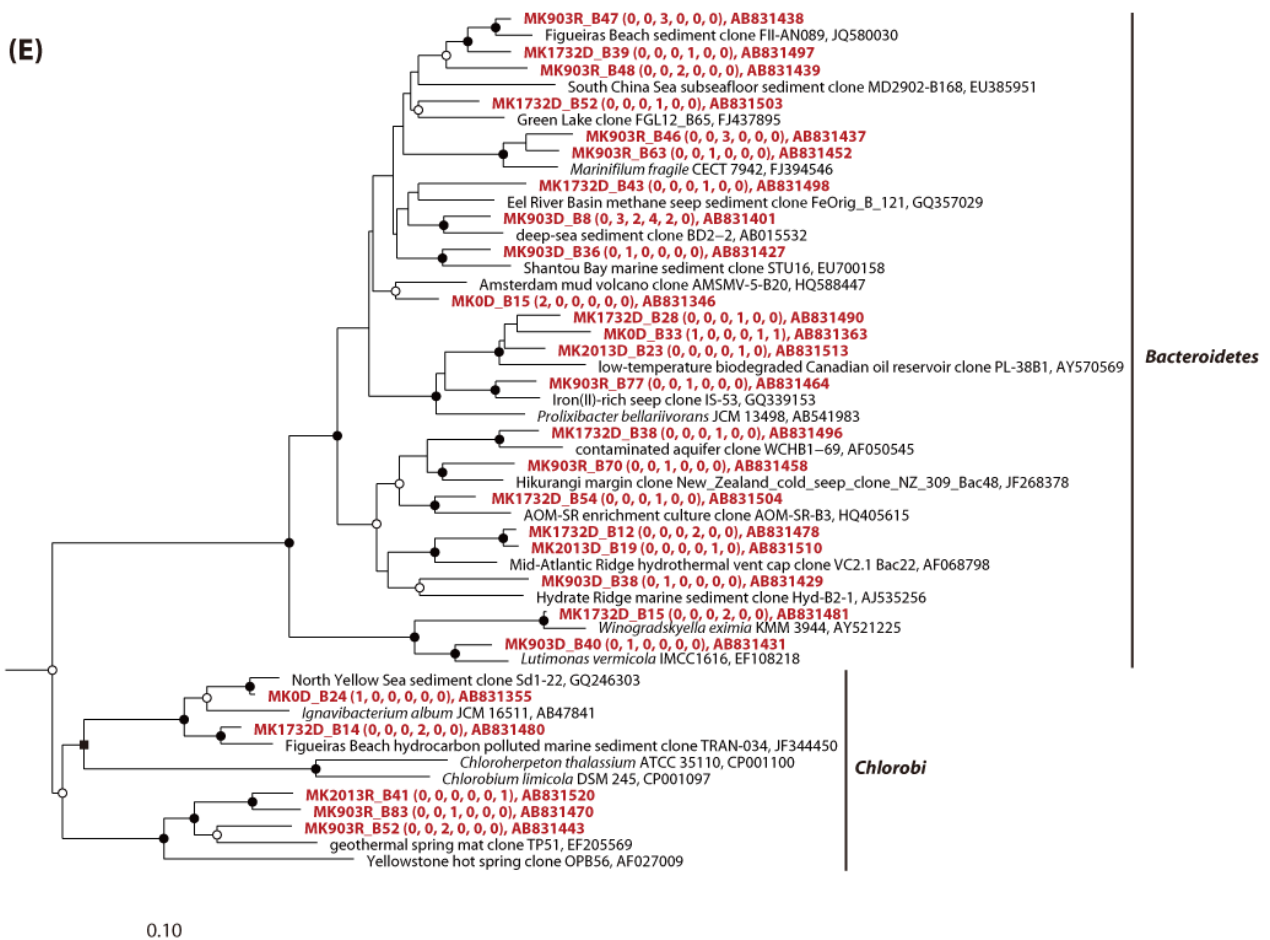Figure S5. Aoki *et al.*

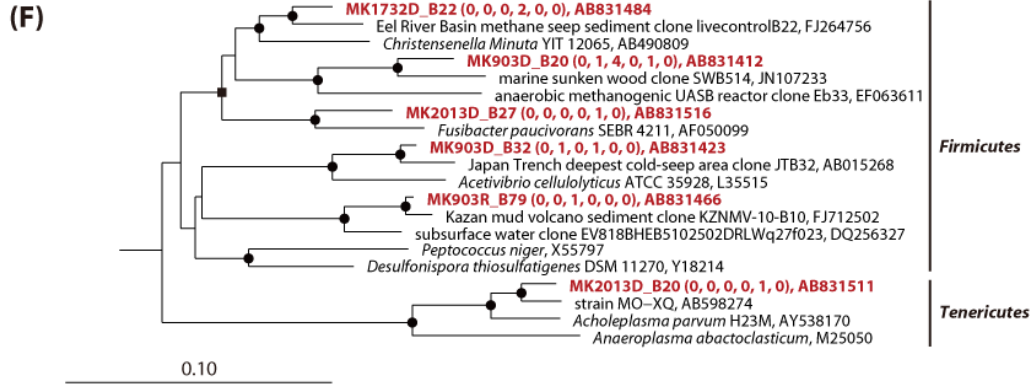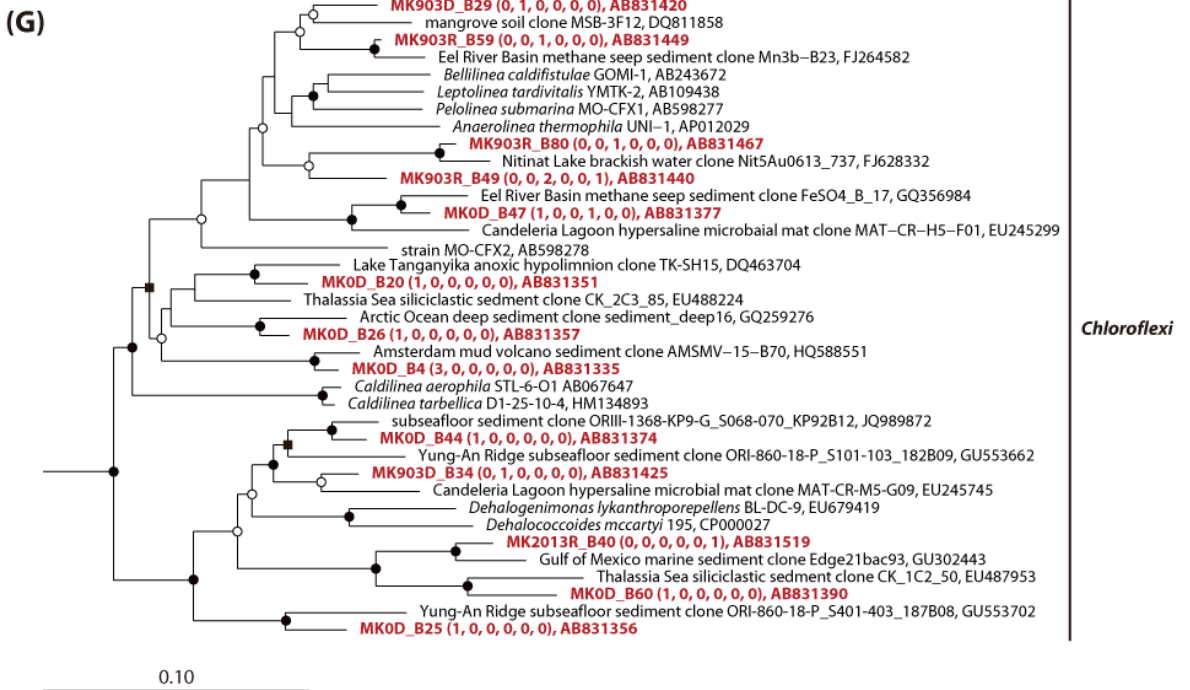

Figure S5. Aoki *et al.*

Supplement: Figure S5 — Phylogenetic tree showing the phylogenetic affiliations of bacterial 16S rRNA gene and 16S rRNA phylotypes obtained in this study. The initial tree was constructed with sequences longer than 1,200 nucleotides, using the neighbor-joining method. Shorter sequences were subsequently inserted into the tree using the parsimony insertion tool in the ARB program. Three archaeal sequences (Methanosarcina acetivorans C2A [AE010299], Thermococcus profundus DT5432 [Z75233], and Nitrosopumilus maritimus SCM1 [CP000866]) were used as the outgroups (not shown). (A) A large bacterial tree including diverse bacterial groups. (B–G) Expanded bacterial phylogenetic trees for (B) Deltaproteobacteria, (C) Alphaproteobacteria, Betaproteobacteria, Gammaproteobacteria, and Epsilonproteobacteria, (D) Verrucomicrobia, Lentisphaerae, Chlamidiae, and Planctomycetes, (E) Bacteroidetes and Chlorobi, (F) Firmicutes and Tenericutes, and (G) Chloroflexi. The scale bars represent the estimated number of nucleotide changes per sequence position. The bold and colored sequences, symbols at the nodes, and numbers in the parentheses indicate the same meanings as in Fig. S3. (PDF) [file pone.0105356.s005.pdf]

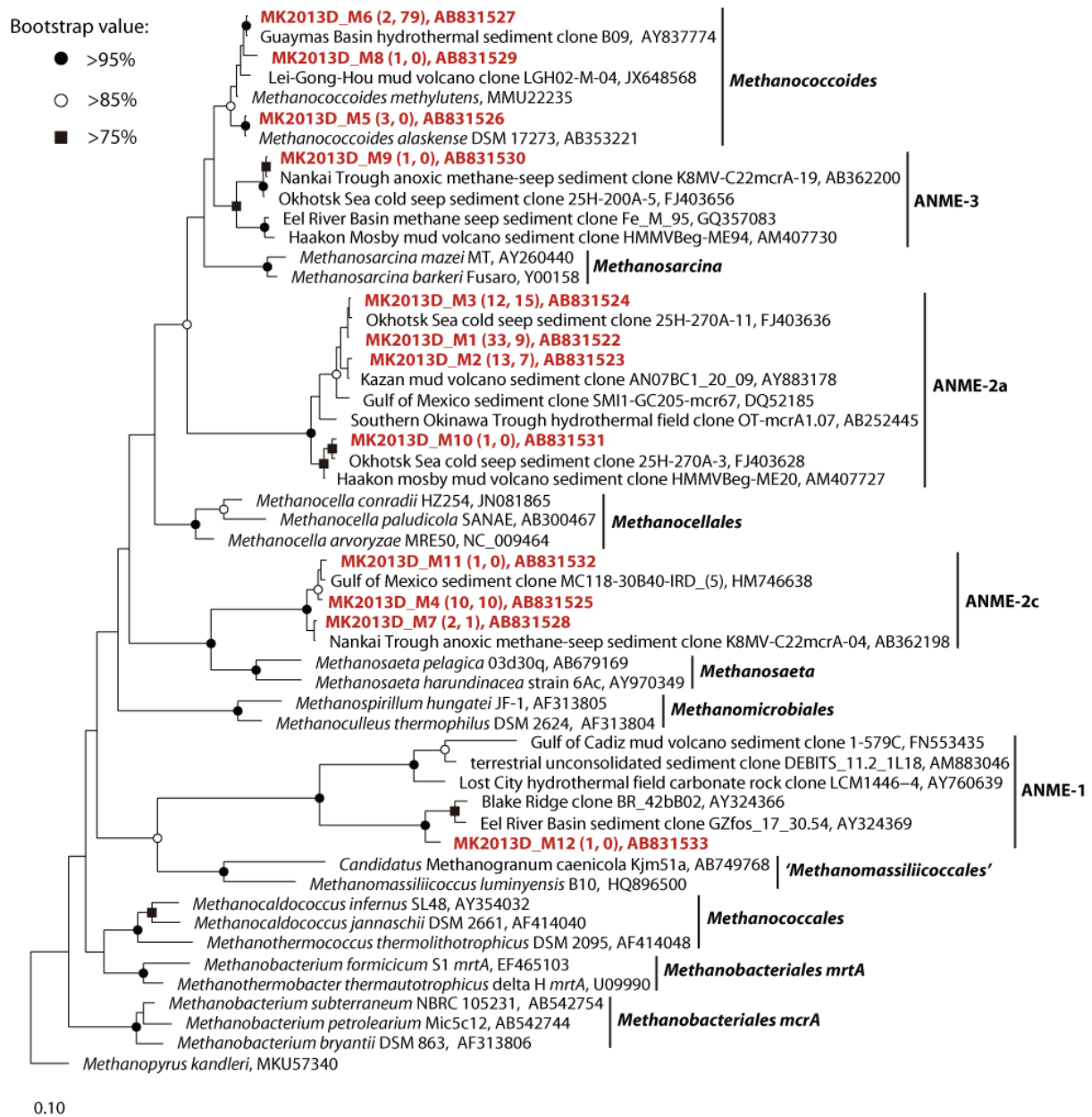

Figure S6. Aoki *et al.*

Supplement: Figure S6 — Phylogenetic tree showing the phylogenetic affiliations of deduced McrA amino acid sequences obtained in this study. The numbers in parentheses indicate the number of phylotypes in each clone library and their frequency in each library in the following order: mcrA gene-based clone library at day 2,013, and mcrA mRNA-based clone library at day 2,013. The scale bar indicates 10% estimated sequence divergence. The meanings of the bold and colored sequences, and symbols at the nodes are the same as in Fig. S3. (PDF) [file pone.0105356.s006.pdf]

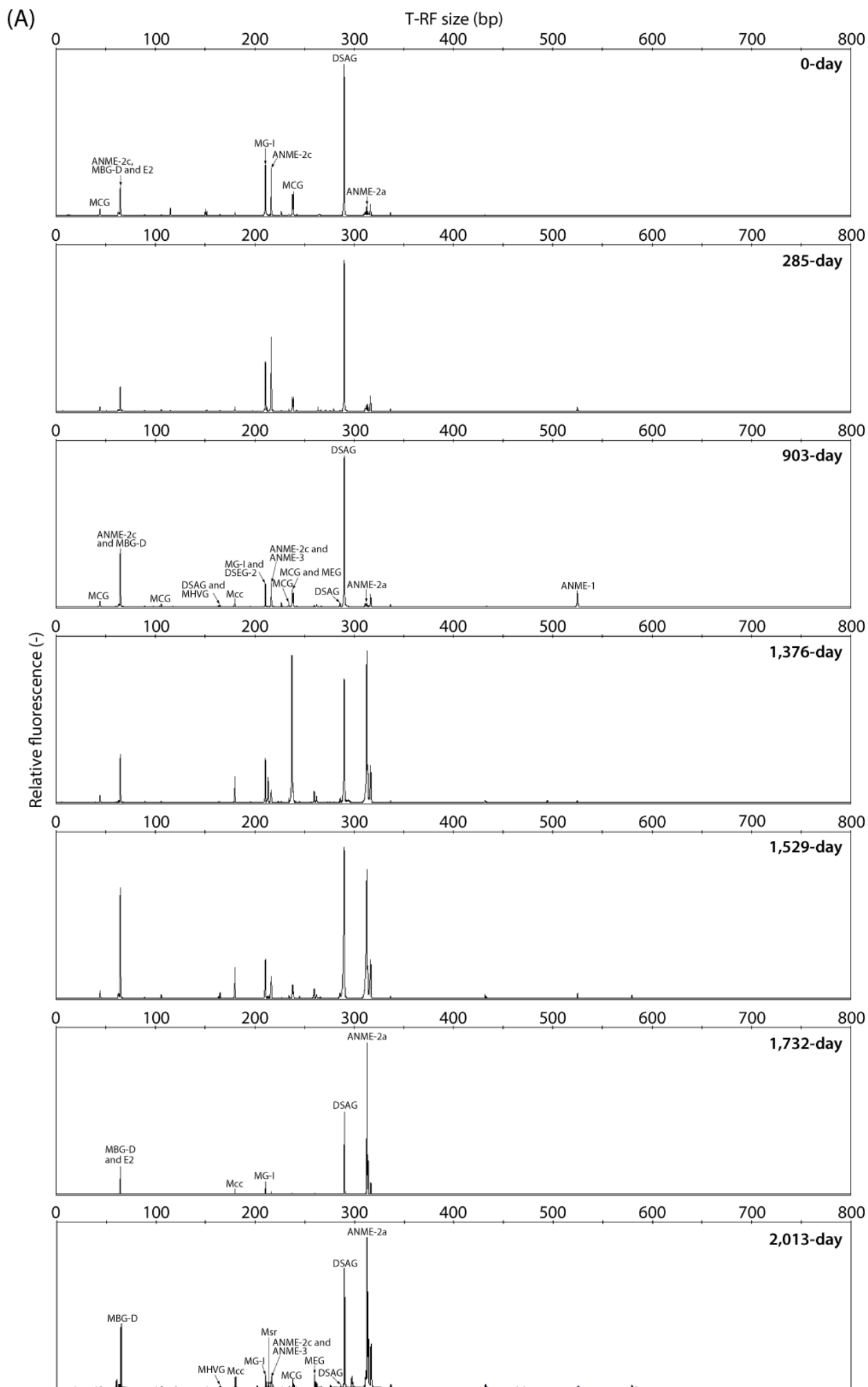

**Figure S7.** Aoki *et al.*

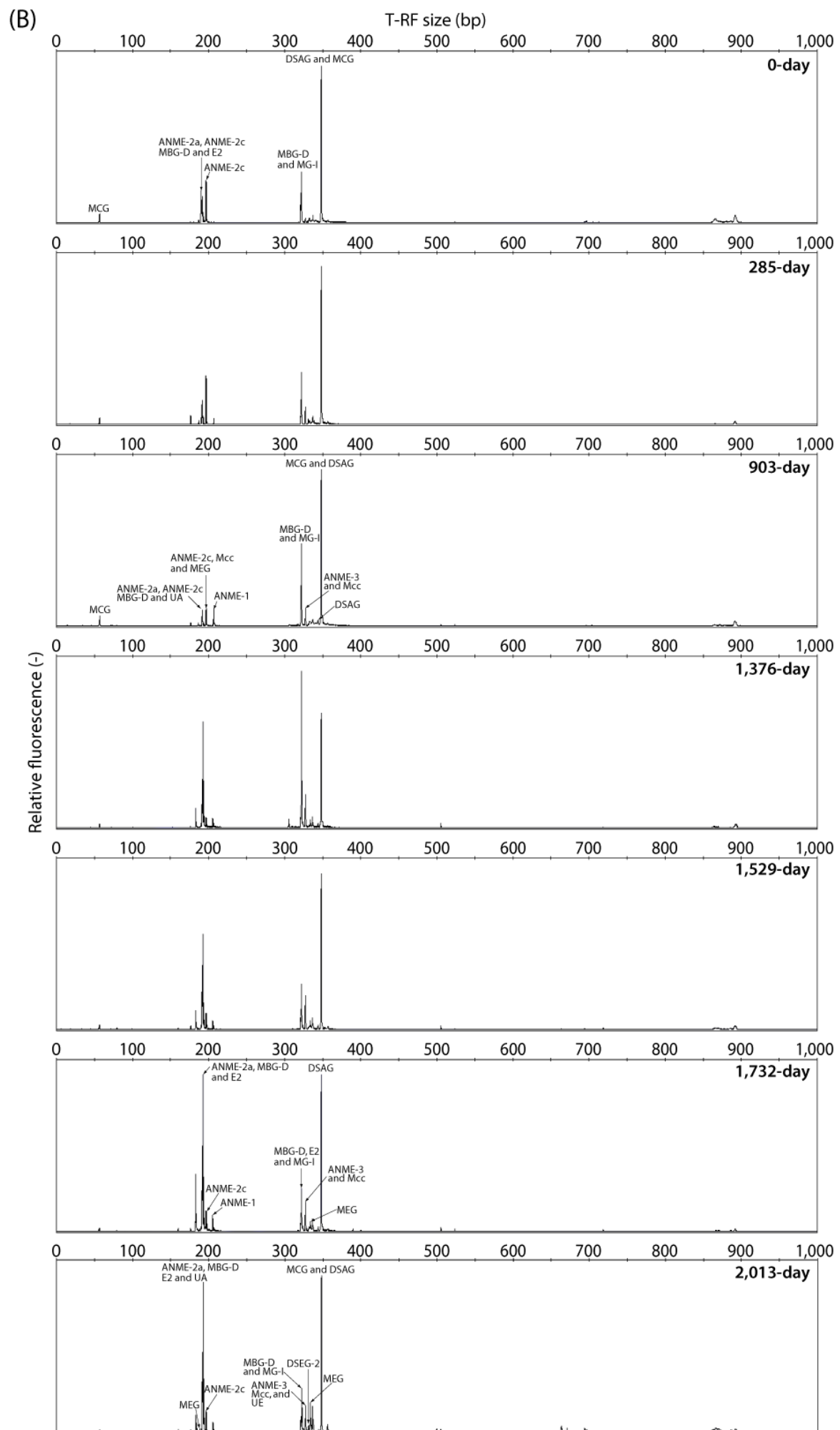

Figure S7. Aoki *et al.*

Supplement: Figure S7 — T-RFLP profiles of archaeal 16S rRNA genes digested with (A) HaeIII or (B) HhaI. The phylogenetic affiliations of each T-RF were identified using the archaeal 16S rRNA gene and 16S rRNA clone sequences obtained in this study. The abbreviations for some peaks are as follows: Mcc, Methanococcoides; Msr, uncultured Methanosarcinaceae; UE, unclassified Euryarchaeota; and UA, unclassified Archaea. (PDF) [file pone.0105356.s007.pdf]

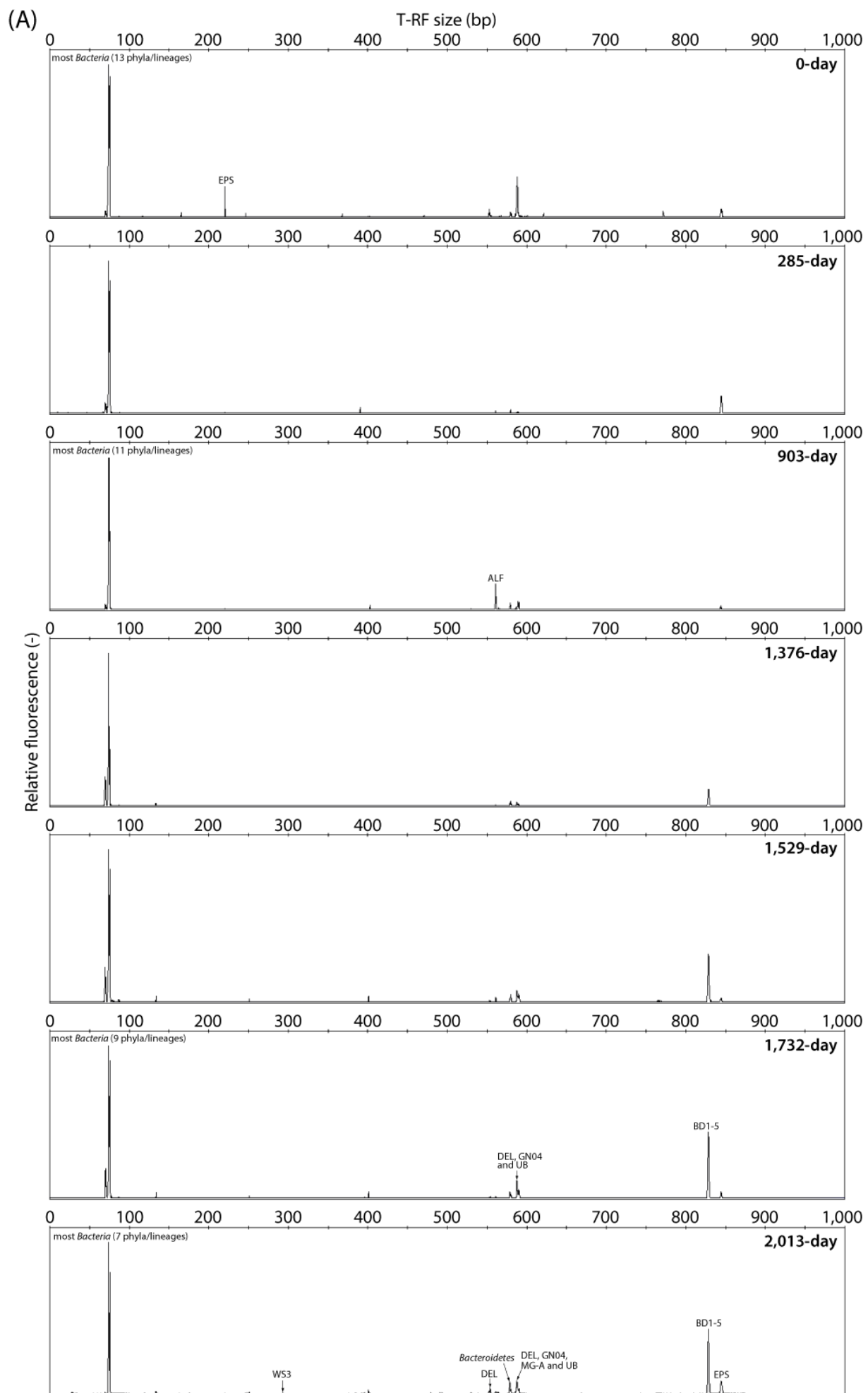

**Figure S8.** Aoki *et al.*

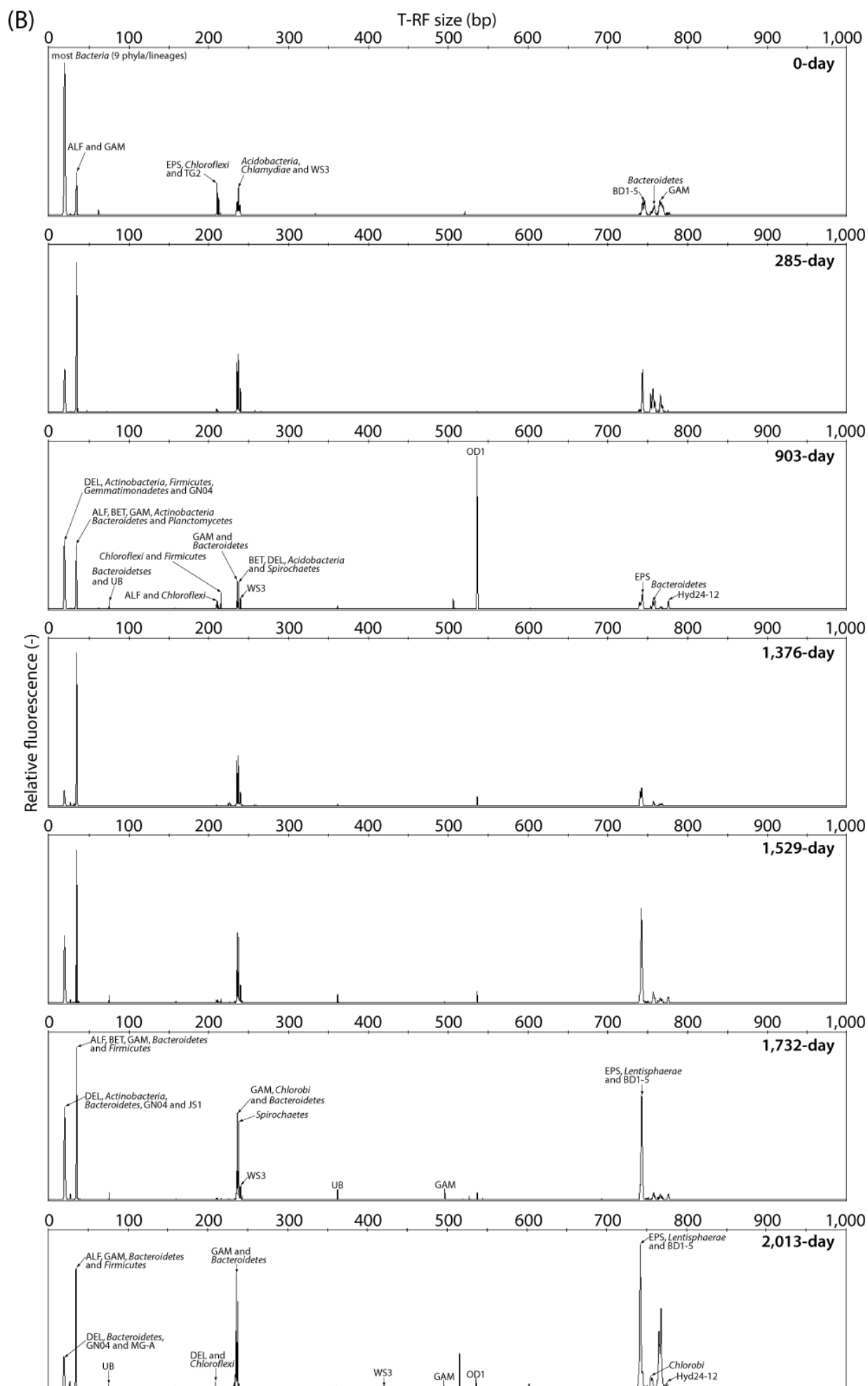

Figure S8. Aoki *et al.*

Supplement: Figure S8 — T-RFLP profiles of bacterial 16S rRNA genes digested with (A) HaeIII or (B) HhaI. The phylogenetic affiliations of each T-RF were identified using the bacterial 16S rRNA gene and 16S rRNA clone sequences obtained in this study. The abbreviations for some peaks are as follows: ALF, Alphaproteobacteria; BET, Betaproteobacteria; GAM, Gammaproteobacteria; DEL, Deltaproteobacteria; EPS, Epsilonproteobacteria; and UB, unclassified Bacteria. (PDF) [file pone.0105356.s008.pdf]

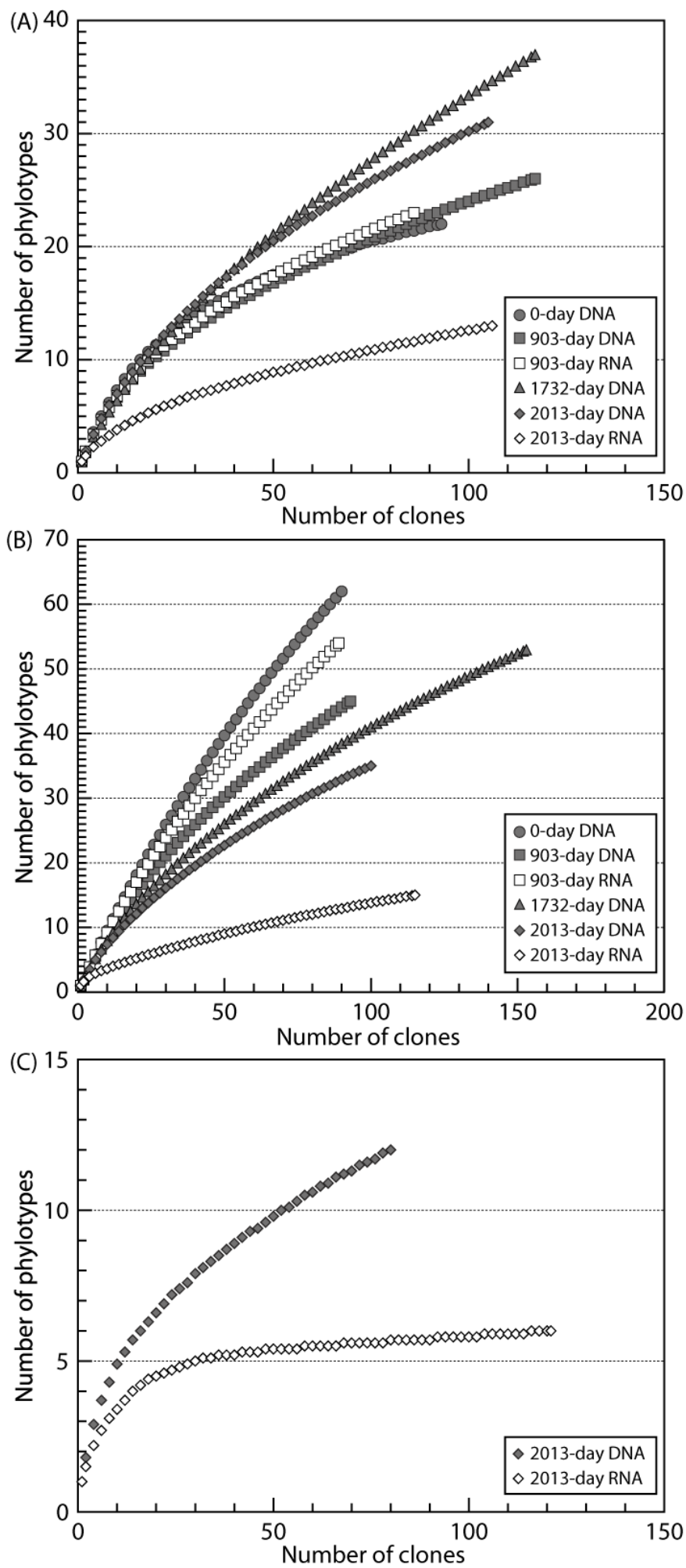

**Figure S9.** Aoki *et al.*

Supplement: Figure S9 — Rarefaction curves for (A) archaeal and (B) bacterial 16S rRNA genes and 16S rRNA, and (C) mcrA genes and mRNA. (PDF) [file pone.0105356.s009.pdf]
