# Supplementary material for: A Long-Term Cultivation of an Anaerobic Methane-Oxidizing Microbial Community from Deep-Sea Methane-Seep Sediment Using a Continuous-Flow Bioreactor
Source: PLoS One. 2014 Aug 20;9(8):e105356. doi: 10.1371/journal.pone.0105356 (PMC4139340; doi:10.1371/journal.pone.0105356)
Supplement: Table S1 — Oligonucleotide primers used in this study. (PDF) [file pone.0105356.s010.pdf]

Table S1 Oligonucleotide primers used in this study.

primer	Target	Primer sequence (5' to 3')	Reference
Arch21F	Archaeal 16S rRNA gene	TTC CGG TTG ATC CYG CCG GA	[1]
340F[a]	Archaeal 16S rRNA gene	CCY TAY GRG GYG CAS CAG G	[2]
Ar912r[a]	Archaeal 16S rRNA gene	CCC CCG CCA ATT CCT TTA A	[3, 4]
932R	Archaeal 16S rRNA gene	GCY CYC CCG CCA ATT CMT TTA	[5]
27F	Bacterial 16S rRNA gene	AGA GTT TGA TCM TGG CTC AG	[6]
EUB338F[b]	Bacterial 16S rRNA gene	ACT CCT ACG GGA GGC AGC	[7]
		ACT CCT ACG GGA GGC TGC	[8]
		ACA CCT ACG GGT GGC TGC	[8]
		ACA CCT ACG GGT GGC AGC	[8]
907R[a]	Bacterial 16S rRNA gene	CCG TCA ATT CMT TTR AGT T	[6]
1492R[a]	Archaeal and bacterial 16S rRNA gene	GGH TAC CTT GTT ACG ACT T	[6]
MLf	*mcrA* gene	GGT GGT GTM GGA TTC ACA CAR TAY GCW ACA GC	[9]
MLr	*mcrA* gene	TTC ATT GCR TAG TTW GGR TAG TT	[9]

[a]These primers are a slightly modified version of the original designed primers.

[b]This primer consisted of a mixture of four primers at an equal amount (mol).

References

1. DeLong, EF (1992) Archaea in coastal marine environments. Proc. Natl. Acad. Sci. U. S. A. 89:5685–5689.
2. Gantner, S, Andersson, AF, Alonso-Sáez, L, Bertilsson, S (2011) Novel primers for 16S rRNA-based archaeal community analyses in environmental samples. J. Microbiol. Methods 84:12–18.
3. Großkopf, R, Janssen, PH, Liesack, W (1998) Diversity and structure of the methanogenic community in anoxic rice paddy soil microcosms as examined by cultivation and direct 16S rRNA gene sequence retrieval. Appl. Environ. Microbiol. 64:960–969.
4. Lueders, T, Friedrich, M (2000) Archaeal population dynamics during sequential reduction processes in rice field soil. Appl. Environ. Microbiol. 66:2732–2742.
5. Murakami, S, Fujishima, K, Tomita, M, Kanai, A (2012) Metatranscriptomic analysis of microbes in an Oceanfront deep-subsurface hot spring reveals novel small RNAs and type-specific tRNA degradation. Appl. Environ. Microbiol. 78:1015–1022.
6. Lane DJ (1991) 16S/23S rRNA sequencing. In: Stackebrandt E, Goodfellow M, editors. Nucleic acid techniques in bacterial systematics. New York, NY: John Wiley & Sons. pp. 115-175.
7. Amann, RI, Binder, BJ, Olson, RJ, Chisholm, SW, Devereux, R, et al. (1990) Combination of 16S rRNA-targeted oligonucleotide probes with flow cytometry for analyzing mixed microbial populations. Appl. Environ. Microbiol. 56:1919–1925.
8. Daims, H, Brühl, A, Amann, R, Schleifer, K-H, Wagner, M (1999) The domain-specific probe EUB338 is insufficient for the detection of all *Bacteria*: development and evaluation of a more comprehensive probe set. Syst. Appl. Microbiol. 22:434–444.
9. Luton, PE, Wayne, JM, Sharp, RJ, Riley, PW (2002) The *mcrA* gene as an alternative to 16S rRNA in the phylogenetic analysis of methanogen populations in landfill. Microbiology 148:3521–3530.
