# Supplementary material for: A Long-Term Cultivation of an Anaerobic Methane-Oxidizing Microbial Community from Deep-Sea Methane-Seep Sediment Using a Continuous-Flow Bioreactor
Source: PLoS One. 2014 Aug 20;9(8):e105356. doi: 10.1371/journal.pone.0105356 (PMC4139340; doi:10.1371/journal.pone.0105356)
Supplement: Table S2 — 16S rRNA-targeted oligonucleotide probes used in this study. (PDF) [file pone.0105356.s011.pdf]

Table S2 16S rRNA-targeted oligonucleotide probes used in this study.

Probe	Target group	Probe sequence (5' to 3')	%FA[f]	Reference
ARC915	Most *Archaea*	GTG CTC CCC CGC CAA TTC CT	35 (60)	[1]
ANME-1-350	ANME-1	AGT TTT CGC GCC TGA TGC	20[g]	[2]
ANME-2a-647[a]	ANME-2a	TCT TCC GGT CCC AAG CCT	10[g] (50)[g]	[3]
ANME-2c-760[b]	ANME-2c	CGC CCC CAG CTT TCG TCC	35[g] (65)[g]	[3]
ANME-3-1249[c]	ANME-3	TCG GAG TAG GGA CCC ATT	20[h]	[4]
ANME-3-1249H3	Helper probe for ANME-3-1249	GTC CCA ATC ATT GTA GCC GGC		[5]
ANME-3-1249H5	Helper probe for ANME-3-1249	TTA TGA GAT TAC CAT CTC CTT		[5]
MBGD-318[a]	MBG-D	GAT ATC GTG TCT CAG ATA	5[g] (15)[g]	[6]
MBGB-380[d]	DSAG	GTA ACC CCG TCA CAC TTT	10[g] (30)[g]	[3]
MCOCID442[e]	*Methanococcoides*	ACA CAT GCC GTT TAC ACA TG	20	[6]
EUB338	Most *Bacteria*	GCT GCC TCC CGT AGG AGT	20 (20)	[7]
Mγ669	*Methylobacter* and *Methylomonas*	GCT ACA CCT GAA ATT CCA CTC	20	[8]
UncGam731	Gammaproteobacterial phylotype MK903D_B5	AAT GTT AAC CCA GAC AGT CGC	20[g]	This study
NON338	Negative control for CARD-FISH	ACT CCT ACG GGA GGC AGC	(15–65)	[9]

[a]Permeabilization pretreatment with 0–2 μg ml^{-1} proteinase K (room temperature, 10 min) was used for CARD-FISH.

[b]Permeabilization pretreatment with 0.1–1.0 M HCl (room temperature, 1 min) was used for CARD-FISH.

[c]Used together with the unlabeled helper probes ANME-3-1249H3 and ANME-3-1249H5.

[d]Permeabilization pretreatment with 100–150 μg ml^{-1} proteinase K (room temperature, 10 min) was used for CARD-FISH.

[e]To avoid *Methanococcoides* cell losses, the sodium dodecyl sulfate concentration in hybridization and washing buffers was lowered from 0.01 to 0.0001% when using the probe MCOCID442.

[f]Numbers in parentheses are the formamide (FA) concentration of hybridization and washing buffers in CARD-FISH.

[g]These FA concentarions were determined by Clone-FISH [10].

[h]Since a nearly full-length of ANME-3 16S rRNA gene sequence for Clone-FISH was not retrieved from the 903-day sample, the optimal FA concentration reported by Lösekann *et al*. [5] was used.

References

1. Stahl DA, Amann R (1991) Development and application of nucleic acid probes. In: Stackebrandt E, Goodfellow M, editors. Nucleic acid techniques in bacterial systematics. Chichester, UK: John Wiley & Sons. pp. 205–248.
2. Boetius, A, Ravenschlag, K, Schubert, CJ, Rickert, D, Widdel, F, et al. (2000) A marine microbial consortium apparently mediating anaerobic oxidation of methane. Nature 407:623–626.
3. Knittel, K, Lösekann, T, Boetius, A, Kort, R, Amann, R (2005) Diversity and distribution of methanotrophic archaea at cold seeps. Appl. Environ. Microbiol. 71:467–479.
4. Niemann, H, Lösekann, T, de Beer, D, Elvert, M, Nadalig, T, et al. (2006) Novel microbial communities of the Haakon Mosby mud volcano and their role as a methane sink. Nature 443:854–858.
5. Lösekann, T, Knittel, K, Nadalig, T, Fuchs, B, Niemann, et al. (2007) Diversity and abundance of aerobic and anaerobic methane oxidizers at the Haakon Mosby Mud Volcano, Barents Sea. Appl. Environ. Microbiol. 73:3348–3362.
6. Imachi, H, Aoi, K, Tasumi, E, Saito, Y, Yamanaka, Y, et al. (2011) Cultivation of methanogenic community from subseafloor sediments using a continuous-flow bioreactor. ISME J 5:1913–1925.
7. Amann, RI, Binder, BJ, Olson, RJ, Chisholm, SW, Devereux, R, et al. (1990) Combination of 16S rRNA-targeted oligonucleotide probes with flow cytometry for analyzing mixed microbial populations. Appl. Environ. Microbiol. 56:1919–1925.
8. Eller, G, Stubner, S, Frenzel, P (2001) Group-specific 16S rRNA targeted probes for the detection of type I and type II methanotrophs by fluorescence in situ hybridisation. FEMS Microbiol. Lett. 198:91–97.
9. Wallner, G, Amann, R, Beisker, W (1993) Optimizing fluorescent in situ hybridization with rRNA-targeted oligonucleotide probes for flow cytometric identification of microorganisms. Cytometry 14:136–143.
10. Schramm, A, Fuchs, BM, Nielsen, JL, Tonolla, M, Stahl, DA (2002) Fluorescence *in situ* hybridization of 16S rRNA gene clones (Clone-FISH) for probe validation and screening of clone libraries. Environ. Microbiol. 4:713–720.
