# Supplementary material for: A Long-Term Cultivation of an Anaerobic Methane-Oxidizing Microbial Community from Deep-Sea Methane-Seep Sediment Using a Continuous-Flow Bioreactor
Source: PLoS One. 2014 Aug 20;9(8):e105356. doi: 10.1371/journal.pone.0105356 (PMC4139340; doi:10.1371/journal.pone.0105356)
Supplement: Table S3 — Summary of FISH and CARD-FISH results. (PDF) [file pone.0105356.s012.pdf]

Table S3 Summary of FISH and CARD-FISH results.

Probe	Target group	Sample/Detection method			
		903-day sample		2,013-day sample	
		FISH	CARD-FISH	FISH	CARD-FISH
ARC915	Most *Archaea*	+	+	+	+
ANME-1-350	ANME-1	+	NT	+	NT
ANME-2a-647	ANME-2a	-	+	-	+
ANME-2c-760	ANME-2c	-	+	-	-
ANME-3-1249	ANME-3	-	NT	-	NT
MBGD-318	MBG-D	-	+	-	+
MBGB-380	DSAG	-	+	-	+
MCOCID442	*Methanococcoides*	+	NT	+	NT
EUB338	Most *Bacteria*	+	+	+	+
Mγ669	*Methylobacter* and *Methylomonas*	+	NT	+	NT
UncGam731	Gammaproteobacterial phylotype MK903D_B5	+	NT	+	NT

+: detected, -: not detected, NT: not tested.
